# The signalling receptor MCAM coordinates apical-basal polarity and planar cell polarity during morphogenesis

**DOI:** 10.1038/ncomms15279

**Published:** 2017-06-07

**Authors:** Qian Gao, Junfeng Zhang, Xiumei Wang, Ying Liu, Rongqiao He, Xingfeng Liu, Fei Wang, Jing Feng, Dongling Yang, Zhaoqing Wang, Anming Meng, Xiyun Yan

**Affiliations:** 1 Key Laboratory of Protein and Peptide Pharmaceuticals, Institute of Biophysics, Chinese Academy of Sciences, Beijing 100101, China; 2 University of Chinese Academy of Sciences, Beijing 100049, China; 3 State Key Laboratory of Biomembrane and Membrane Engineering, Tsinghua-Peking Center for Life Sciences, Tsinghua University, Beijing 100084, China; 4 State Key Laboratory of Brain and Cognitive Science, Institute of Biophysics, Chinese Academy of Sciences, Beijing 100101, China; 5 Key Laboratory of Mental Health, Institute of Psychology, Chinese Academy of Sciences, Beijing 100101, China

## Abstract

The apical–basal (AB) polarity and planar cell polarity (PCP) provide an animal cell population with different phenotypes during morphogenesis. However, how cells couple these two patterning systems remains unclear. Here we provide *in vivo* evidence that melanoma cell adhesion molecule (MCAM) coordinates AB polarity-driven lumenogenesis and c-Jun N-terminal kinase (JNK)/PCP-dependent ciliogenesis. We identify that MCAM is an independent receptor of fibroblast growth factor 4 (FGF4), a membrane anchor of phospholipase C-γ (PLC-γ), an immediate upstream receptor of nuclear factor of activated T-cells (NFAT) and a constitutive activator of JNK. We find that MCAM-mediated vesicular trafficking towards FGF4, while generating a priority-grade transcriptional response of NFAT determines lumenogenesis. We demonstrate that MCAM plays indispensable roles in ciliogenesis through activating JNK independently of FGF signals. Furthermore, *mcam*-deficient zebrafish and *Xenopus* exhibit a global defect in left-right (LR) asymmetric establishment as a result of morphogenetic failure of their LR organizers. Therefore, MCAM coordination of AB polarity and PCP provides insight into the general mechanisms of morphogenesis.

## Abstract (web summary)

It is unclear if there is an interaction between apical-basal (AB) polarity and planar cell polarity (PCP) pathways during morphogenesis. Here, the authors define a role for the melanoma cell adhesion molecule (MCAM), known to regulate PCP, in AB polarity via FGF signalling *in vitro* and in zebrafish.

During morphogenesis, apical–basal (AB) polarity and planar cell polarity (PCP) are prerequisites for organizing cells into accurate structures such as tissues and organs^1^,^2^. These two distinct patterning systems are composed of two different sets of proteins, but both concurrently operate during morphogenesis^3^,^4^,^5^. However, the major question in morphogenesis, how cells couple these two polarity systems to simultaneously develop different morphologies, remains unclear^1^,^2^,^5^.

Formation of multicellular rosette-like structures is a common intermediate process during morphogenesis in diverse species and is a well-studied example of cellular polarity-based processes^6^,^7^. The zebrafish LR organizer of Kupffer's vesicle (KV) and the posterior lateral line (PLL) system are typical rosette structures^5^. Like adult cells in vertebrate, rosette cells also have ubiquitous organelles of cilia. Each rosette-like structure arises from the proliferation of a group of ciliated cells to form a spherical architecture of polarized cells enclosing a central lumen^5^. Lumenogenesis requires AB polarity to dictate correct cellular arrangements^8^ and ciliogenesis needs JNK/PCP to regulate cilia assembly^4^.

MCAM, also called CD146 or MUC18, is a signalling receptor and exerts its physiological functions primarily on embryonic development^9^. However, our understanding of how MCAM regulates morphogenesis remains poorly understood. It has been well documented that the polarized distribution of MCAM on the leading edge of chemotaxing cells is vital for establishing cell polarity, but the underlying mechanisms are unclear^10^. In these polarized cells, the concentration of calcium ions (Ca^2+^) is spatially enhanced and polarized in the MCAM-enriched region^11^. Ca^2+^ is a crucial signalling element for activation of NFAT, a Ca^2+^-dependent transcriptional response required for vertebrate development^12^.

FGF signalling plays indispensable roles in the development of living organisms and primarily activates transcriptional factors for activator protein 1 (AP-1), forkhead box protein (FOXO) and NFAT^13^. The chief intracellular substrates downstream from FGF signalling are FGFR substrate 2 (FRS2) and PLC-γ. Upon FGF signals, FRS2 switches on AP-1 and FOXO, while PLC-γ turns on NFAT. The functional parallels between MCAM and FGF signalling in diverse processes, such as neural patterning, stem cell maintenance, angiogenesis, wound healing and epithelial–mesenchymal transition (EMT)^9^,^14^,^15^, hint that an unknown interplay between these two pathways exists.

The zebrafish KV plays central roles in LR asymmetric patterning and is an ideal model for investigation into the epithelial organ morphogenesis of metazoan development^16^. Inspired by the involvement of FGF signalling in the morphogenesis of vertebrate LR organizer and in LR patterning^17^, we hypothesized that MCAM may also exert similar morphogenetic functions as FGF signalling. Here, we have validated this hypothesis at *in vitro* and *in vivo* levels. We demonstrate that MCAM coordinates two distinct patterning systems of AB polarity and PCP, mechanistically explaining how cell populations develop distinct morphologies synchronously. To achieve this coordination, MCAM concurrently activates NFAT and JNK to control AB polarity and PCP, respectively. Thus, our study suggests that MCAM coordination of AB polarity and JNK/PCP is a general mechanism for morphogenesis of organs/tissues during development.

## Results

### MCAM is an independent receptor of FGF4

MCAM is required for diverse developmental events and is a developmentally regulated signalling receptor with higher abundance in tissues/organs during embryogenesis relative to an adult stage^9^. To decipher the mechanisms underlying MCAM functions in development, we sought to identify MCAM ligands that are necessary and sufficient for morphogenesis. We therefore performed a yeast two-hybrid assay by using the MCAM-GAL4-binding domain fusion protein as the bait to screen a human GAL4-activating domain fusion complementary DNA (cDNA) library.

**Figure 1:**
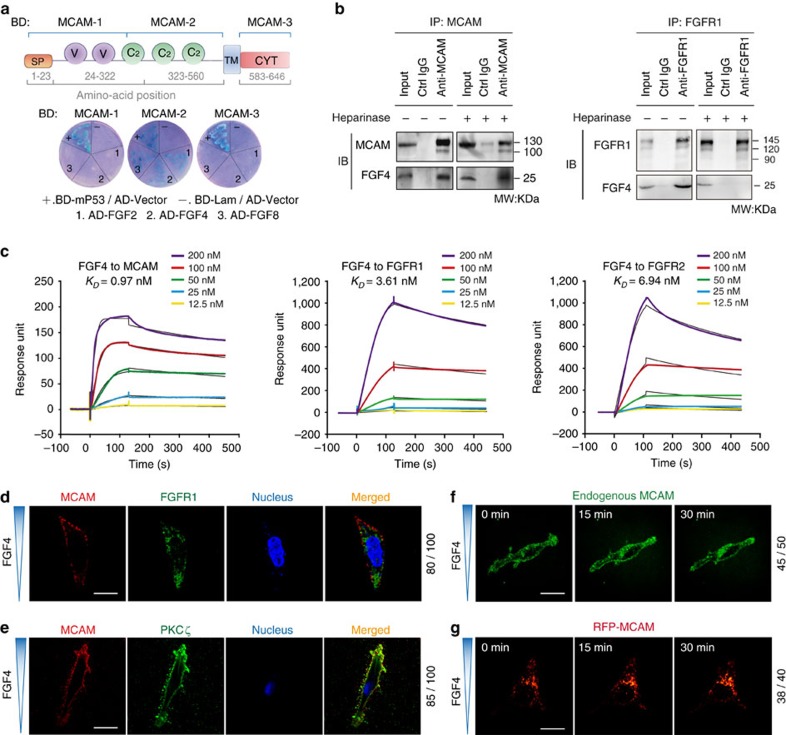
MCAM/FGF4-dependent apical surface biogenesis. (**a**) Upper panel, cartoon of MCAM-BD 1–3. Lower panel, yeast zygotes obtained after mating the bait strain containing pGBKT7-MCAM with the library strain containing pGADT7-FGF2, FGF4, and FGF8. BD, DNA-binding domain; AD, activation domain; +, positive control with p53-BD; and −, negative control with empty AD vector and BD-lambda. (**b**) Co-immunoprecipitation of MCAM/FGF4 and FGFR1/FGF4 with the protein lysate treated with or without the heparinase I and heparinase III (0.06 IU ml^−l^). (**c**) Kinetic dissociation constant (*K*_D_) of FGF4/MCAM, FGF4/FGFR1, or FGF4/ FGFR2 complexes was measured using a surface plasmon resonance method. (**d**) Distribution of polarized MCAM and unpolarized FGFR1 on chemotaxing cells. The source concentration of FGF4 in the chemotaxis assay is 10 ng ml^−l^. (**e**) Co-localization of endogenous MCAM and the apical marker aPKCζ in chemotaxing cells. Scale bar, 20 μm. (**f**,**g**) Time-lapse live-cells imaging of endogenous MCAM (**f**) or exogenous MCAM-RFP (**g**) at the leading edge of chemotaxing cells.

The transmembrane domain of MCAM was deleted in the constructs to ensure nuclear localization of the MCAM bait proteins. The extra- or intra-cellular domains of MCAM were inserted in-frame into the pGBKT7 expression vector to generate MCAM-1, MCAM-2 and MCAM-3 constructs (Fig. 1a; Supplementary Fig. 1). More than 1,000 prey were sequenced and analysed by BLAST searches, and an array of in-frame coded sequences was identified that represented potential MCAM-interacting proteins. Among them, FGF2, 4 and 8 were identified as prey for MCAM-2 (Fig. 1a).

Endogenous levels of MCAM protein are disparate in different cell lines, with high levels in embryonic or cancerous cells and low levels in most normal cell lines^9^. We screened several cell lines to select one suitable for mimicking morphogenetic processes with high endogenous MCAM. The HEK293 cell line exhibits a high endogenous level of MCAM, comparable to that of several tumour cell types, and was thus selected for use in functional experiments of this investigation (Supplementary Fig. 2).

Co-immunoprecipitation (co-IP) experiments verified that FGF4 and MCAM formed a complex in HEK293 cells (Fig. 1b). Notably, none of the FGFRs (FGFR1–4) tested co-immunoprecipitated with MCAM (Supplementary Fig. 2). FGF4 belongs to the paracrine FGFs that signal FGFRs by forming a tripartite complex with FGFRs and heparan sulphate proteoglycan (HSPG)^13^. To examine the potential requirement of HSPGs for interaction of MCAM with FGF4, we performed co-IP with samples of whole cell lysate that were pre-treated with heparinase to degrade endogenous HSPGs. We found heparinase treatment blocked the association of FGF4–FGFR1 but not that of FGF4-MCAM (Fig. 1b). These data thus provide solid evidence that MCAM is a genuine receptor of FGF4 and transmits FGF4 signals independently of HSPG and separately from FGFRs.

To confirm direct interaction and measure the binding affinity of FGF4 to MCAM, a surface plasmon resonance (SPR) assay was conducted. Results showed that FGF4 bound to MCAM with an affinity of 0.97 nM, which was higher than the values observed for FGF4 binding to FGFR1 and FGFR2 (Fig. 1c). FGF2 and 8 did not bind to MCAM although binding to FGFR1 and FGFR2 was detected (Supplementary Fig. 3).

### MCAM mediates vesicular transportation in response to FGF4

FGF4 acts as a chemoattractant during the morphogenesis of chick LR organizer^18^. To evaluate the functions of MCAM or FGFRs on cellular behaviours responding to FGF4, we first conducted chemotaxis assays to mimic the process of cell polarity. FGF2 and 8 were included as controls. Post 1 h addition of FGF2, 4 and 8, MCAM became enriched at the anterior edge of the chemotaxing cells in the direction of the FGF4 gradient. In contrast, FGFR1 was uniformly distributed on the polarized cell membranes in about 80% of observed cells (Fig. 1d). MCAM enrichment cannot be observed in the chemotaxing cells with the addition of FGF2 and 8 (Supplementary Fig. 4). Furthermore, in about 85% of chemotaxing cells, MCAM was co-localized with atypical (a) PKCζ and PAR3 (two apical markers) at the cellular leading edge in the direction of the FGF4 gradient (Fig. 1e; Supplementary Fig. 5). These results indicate that MCAM is a previously unidentified responsive receptor of FGF4 and that MCAM-enriched leading edge is equivalent to the apical surface of luminal organs.

To further understand how MCAM enriched at the anterior edge of the chemotaxing cells responds to FGF4, endogenous and exogenous MCAM and endogenous FGFR1 were tracked by live-cell imaging in a chemotaxis assay. After the addition of FGF4, endogenous MCAM became enriched at the anterior edge of the chemotaxing cells in the direction of the FGF4 gradient after an average time of 30 min in about 90% of tested cells (Fig. 1f; Supplementary Movie 1). Exogenous MCAM-RFP showed similar localization and trafficking patterns within 30 min in about 95% of tested cells. The direction of MCAM^+^ endocytic vesicular transportation was identical to that of cellular movement towards the FGF4 gradient within the period of monitoring (Fig. 1g; Supplementary Movie 2). In contrast, such movement was not observed for endogenous FGFR1 (Supplementary Movie 3). These results demonstrate that the asymmetric distribution of MCAM is mediated through endocytic vesicular transportation.

### Loss of MCAM function impairs lumenogenesis

**Figure 2:**
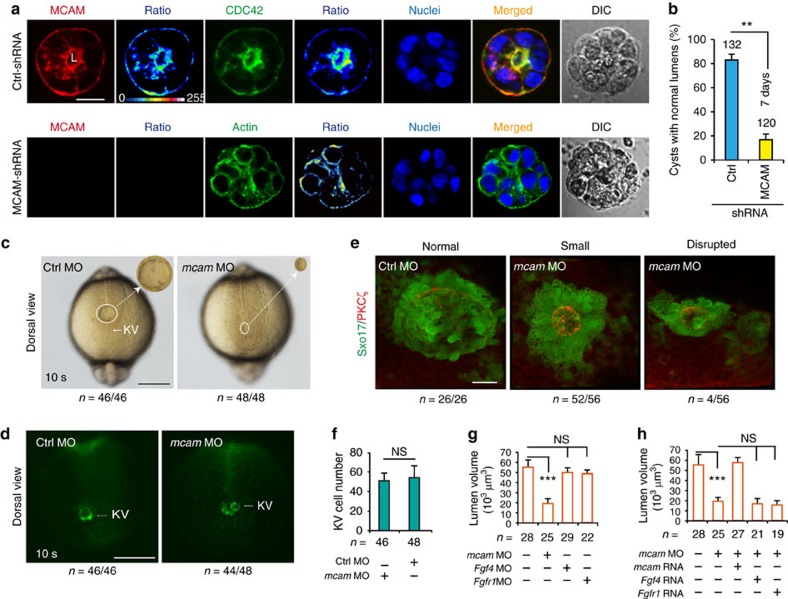
*Mcam* depletion disrupts lumenogenesis. (**a**,**b**) HEK293 cells transfected with either control pGPU6/Neo-shRNA vector or pGPU6/Neo-MCAM-shRNA were cultured on Matrigel for 5–7 days to enable cyst formation. Cyst nuclei and F-actin were labelled with DAPI and phalloidin, respectively. DIC means digital image of contrast. The fluorescent images were converted to 16-colour intensity images indicating the localization of MCAM, CDC42, or F-actin (calibration bar: signal intensity). L, lumen space; Scale bar, 20 μm. Data (mean±s.e.m., *n*=3) were analysed with unpaired Student's *t*-test. **P value<0.01. (**c**,**d**) KV in *Sox17:GFP* zebrafish TG embryos injected with *mcam* MO or control MO into DFC. Embryos were collected at 10 s (somite) stage. In representative light micrographs (**c**), the area with KV was enlarged 4 times and shown as inset in the right hand corner. Scale bar, 250 μm. (**e**) *mcam* MO was injected into DFC of *Sox17:GFP* transgenic embryos. Lumen cells were immunolabeled with aPKC antibody (red). Representative fluorescence images show Sox17-GFP-labelled KV (green) and lumen cells (red). Scale bar, 20 μm. (**f**) KV cell numbers in DFC^*mcam* MO^ embryos. Data (mean±s.e.m., *n*=3) were analysed with unpaired Student's *t*-test and NS, not significant. (**g**) Comparison of KV lumen volumes in embryos with DFC injection of *mcam, fgf4* or *fgfr1* MO. KV lumen volumes were calculated using the measure stack tool of Image J software. Data are presented as mean±s.e.m. (*n*=3). One-way analysis of variance (ANOVA) with Tukey's post-test. ***P value<0.001 and NS, not significant. (**h**) Rescue effects of *mcam, fgf4* or *fgfr1* mRNA on reduction of lumen volumes in DFC^*mcam* MO^ embryos. Data are presented as mean±s.e.m. (*n*=3). One-way ANOVA with Tukey's post-test. ***P value<0.001 and NS, not significant.

The core mechanisms underlying the biogenesis of luminal apical surfaces involve the asymmetric distribution of plasma membrane proteins through vesicular trafficking^19^,^20^. We thus speculated that MCAM is involved in lumenogenesis. To test this hypothesis, we decided to use a three-dimensional (3D) *in vitro* model to mimic the lumenogenesis process from a single HEK293 cell^21^. When cells transfected with MCAM-short hairpin (sh) RNA or control shRNA were plated to form cysts (Supplementary Fig. 6), most cells with control shRNA (85%) could form normal lumens. MCAM was localized at the apical surface of cyst lumens; its distribution pattern was similar with CDC42, a required protein in biogenesis of apical surfaces^22^ and an apical marker for cyst lumens^23^. In contrast, MCAM-shRNA knockdown produced obvious defects in luminal biogenesis, with misshapen lumens and disorganized F-actin (an apical marker for cyst lumens) distribution observed in approximately 83% of cysts (Fig. 2a,b). These data indicate that MCAM is a crucial element for *de novo* synthesis of luminal tissues/organs.

Then, we questioned whether FGF4/MCAM signalling plays *in vivo* roles in morphogenesis. We first chose the zebrafish system as an *in vivo* model because of its high re-productivity and transparent embryos. There are two *mcam* genes in zebrafish, *mcama* and *mcamb*. *Mcamb* was the gene examined since it possesses high sequence similarity with the homologous genes in mammals^24^,^25^,^26^. We initially analysed *mcam* mRNA distribution in zebrafish embryos by whole-mount *in situ* hybridization (WISH).

Results showed that *mcam* expression started around mid-gastrulation and was highly enriched in dorsal forerunner cells (DFCs) at 90% epiboly and bud stages, and in KV from the 1-somite stage to the 10-somite stages. At 21 h post-fertilization (hpf), *mcam* was expressed in the blood vasculature, heart, somites and eye field (Supplementary Fig. 7). The spatiotemporal distribution of *fgf4* mRNA in DFC and KV was similar with that of *mcam*, but differed from that of *fgfr1* (Supplementary Fig. 8), implying that MCAM is a rational partner of FGF4 for KV development.

KV morphogenesis includes ciliogenesis and alignment of ciliated cells into luminal structures^27^. To evaluate the specific roles of the investigated genes on KV morphogenesis, we exclusively knocked-down these genes in DFC/KV, but not in the rest of the embryo, and generated DFC^MO or mRNA^ embryos using the transgenic *Tg(sox17: GFP)* zebrafish line^28^. In this line, the KV can be readily visualized during development owing to the expression of green fluorescent protein (GFP) driven by the promoter of the KV marker *Sox17* (ref. 29). Specific knockdown of *mcam* in DFC/KV caused major morphological defects in the size and shape of the KV at the 10-somite stage (Fig. 2c,d). Labelling the KV lumen with an antibody against atypical protein kinase C (aPKC) confirmed that *mcam* knockdown caused about a 65% decrease in KV luminal volume (5.3 × 10^4^ versus 1.8 × 10^4^ μm^3^; *P*<0.001), without affecting the number of KV cells (Fig. 2e,f). In contrast, knockdown of *fgf4* or *fgfr1* in DFC had no effects on the luminal volume of KV (Fig. 2g). The reduction of luminal volume in DFC^*mcam* MO^ embryos was rescued by injecting *mcam* but not *fgf4* or *fgfr1* mRNA into DFC (Fig. 2h; Supplementary Fig. 9), ruling out the possible contribution of FGF signalling on MCAM-mediated AB polarity-driven lumenogenesis.

### Depletion of *mcam* inhibits ciliogenesis

**Figure 3:**
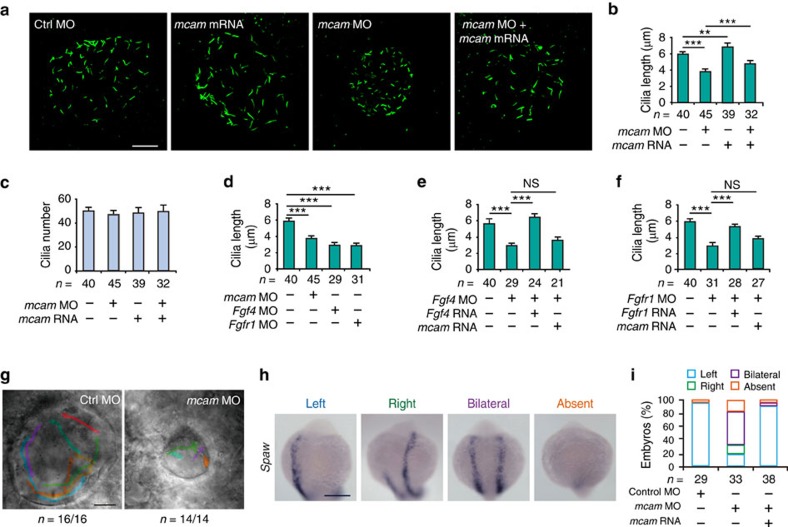
*Mcam* depletion in DFC disrupts ciliogenesis and direction of KV fluid flow in zebrafish. (**a**) KV cilia labelled with an antibody against acetylated tubulin after DFC injection of embryos with control or *mcam* MO, *mcam* mRNA or a combination of *mcam* MO and mRNA. Scale bar, 20 μm. (**b**) Mean cilia length in (**a**) is statistically analysed. Data are presented as mean±s.e.m. One-way ANOVA with Tukey's post-test. **P value<0.01 and ***P value<0.001. (**c**) Mean cilia number (mean±s.e.m.) of embryos in **a** was determined. (**d**) Comparison of cilia length in embryos with DFC injection of *mcam, fgf4* or *fgfr1* MO. Data are presented as mean±s.e.m. One-way ANOVA with Tukey's post-test. ***P value<0.001. (**e**,**f**) Failure of *mcam* mRNA to rescue *fgf4* MO (**e**) or *fgfr1* MO (**f**) induced reduction in cilia length. Data are presented as mean±s.e.m (*n*=3) and analysed using one-way ANOVA with Tukey's post-test. ***P value<0.001 and NS, not significant. (**g**) Direction of KV fluid flow tracked with fluorescent beads in control and DFC^*mcam* MO^ embryos. Scale bar, 20 μm. (**h**) Randomized expression of left side-specific *spaw* in DFC^*mcam* MO^ morphants. Scale bar, 250 μm. (**i**) Quantitative analysis of embryos with normal (left-sided), reversed (right-sided), cardia bifida (bilateral) and absent expression. n, number of embryos (**b**-**f**, **i**).

Depletion of *fgf4* or *fgfr1* causes severe shortening of cilia and the consequent disruption of fluid flow within the KV^17^,^30^,^31^,^32^. Next, we found that the length of KV cilia also decreased by 35% in DFC^*mcam* MO^ embryos relative to DFC^*control* MO^ embryos (3.82 versus 5.85 μm; *P*<0.001). This reduction in cilia length was rescued by DFC injection of *mcam* mRNA into DFC^*mcam* MO^ embryos. In addition, the cilia length was increased by 15% in DFC^*mcam* mRNA^ embryos compared to DFC^*control* MO^ embryos (6.77 versus 5.85 μm; *P*=0.003) (Fig. 3a,b). Consistent with the unchanged KV cell number, these injections did not affect the numbers of cilia (Fig. 3c). The effects of cilia shortening caused by *mcam* knockdown were comparable to those caused by *fgfr1 or fgf4* depletion (Fig. 3d). DFC injection of *mcam* mRNA cannot rescue the defects of cilia shortening in *fgf4* or *fgfr1* DFC-morphants, which was rescued only by DFC-injection of *fgf4* or *fgfr1* mRNA (Fig. 3e,f). However, Mcam overexpression-induced increase of cilia length in wild-type embryos (Fig. 3b) was not found in *fgf4* or *fgfr1* depleted embryos (Fig. 3e,f). Thus, these data suggest that the effects of Mcam on cilia length are not entirely independent of FGF signals.

### Depletion of *mcam* disrupts KV function

Zebrafish KV is a fluid-filled organ and the directional nodal flow in KV plays a central role in controlling LR asymmetric development later on^32^. To observe whether or not Mcam is implicated in KV flow formation, *mcam* knockdowns in DFC were conducted. In contrast to the persistent counter-clockwise movement of fluorescent beads in the KV lumen of control morphants, a consistent directional flow was absent in the KV of DFC^*mcam* MO^ embryos (Fig. 3g; Supplementary Movies 4 and 5).

*Spaw*, the earliest left-sided expressed gene, relays nodal flow-delivered signals in KV to initiate a downstream LR signalling cascade in zebrafish^31^. To assess the position of Mcam in the signalling cascade for LR determination, we analysed the expression of *spaw* and its target genes, including *pitx2*, *lefty1,* and *lefty2* in DFC^*mcam* MO^ embryos. *Mcam* knockdown in DFC perturbed the normal left-sided expression of *spaw* and *pitx2* in the lateral plate mesoderm, and induced the randomized expression of *lefty1 and lefty2,* the markers for the left part of the dorsal diencephalon and heart primordium, respectively (Fig. 3h,i; Supplementary Fig. 10). These data indicate that, similar to FGF signalling, Mcam is also required at an earlier stage of LR development when the nodal flow in KV becomes emergence.

### Mcam plays conserved roles in LR asymmetry development

**Figure 4:**
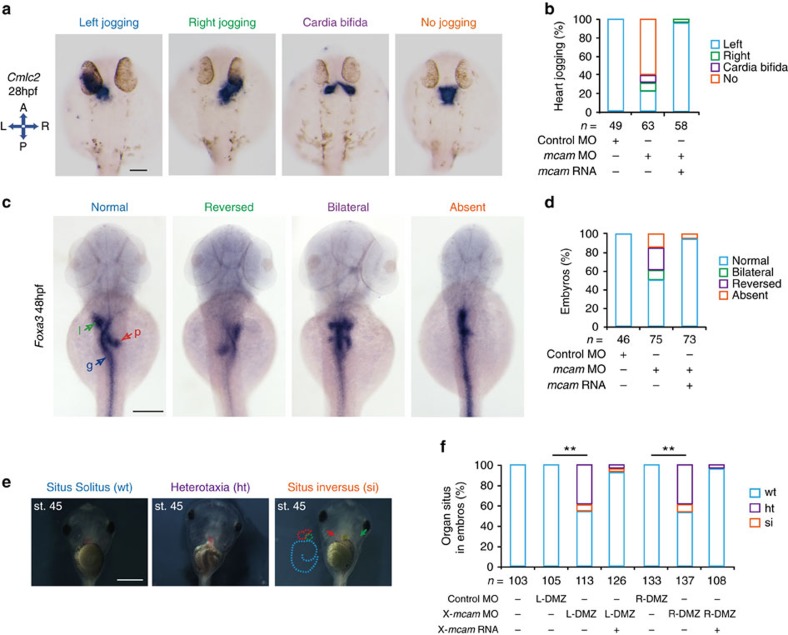
Mcam regulation of left-right asymmetric development in zebrafish and *Xenopus*. (**a**–**d**) Developing organs in DFC^*mcam* MO^ morphants were visualized by WISH using RNA probes of *cmlc2* (**a**, heart) and *foxa3* (**c**, endoderm). Quantitative analysis of embryos with normal, reversed, bilateral and absent asymmetry is shown in **b**,**d**. n, number of embryos. The left schema in **a** shows embryo positions in the embryo (A, anterior; P, posterior; L, left; and R, right). In **c**, l (green arrow)=liver, p (red arrow)=pancreas and g (blue arrow)=gut. H.p.f., hours post-fertilization. Scale bars in **a**,**c** , 100 μm. (**e**) *Xmcam*-MO (1 pM) caused situs defects at stage 45, in *Xenopus* embryos including heterotaxia (ht) and situs inversion (si) compared with the wild type (wt) situs solitus. Heart looping and position is outlined by red dots or red arrow, respectively. Gut coiling is outlined by light blue dots. Position of gall bladder is indicated by green auto-fluorescence and a green arrow. Scale bar, 1 mm. (**f**) Organ situs in Xenopus embryos at tadpole stage injected as specified. Injections were performed in left or right side of dorsal marginal zone (L- or R-DMZ) at the 4-cell stage. Data are presented as mean±s.e.m. Pearson's chi-square tests and **P value<0.01. n, number of embryos.

KV is a transient organ to control LR asymmetric development^32^. To observe effects of Mcam in LR determination, the positions of visceral organs were visualized with heart (*cmlc2*) and liver/pancreas (*foxa3*) markers by WISH. *Mcam* deficiency in DFC caused a severe disruption of cardiac jogging, an early feature of cardiac LR asymmetry, as well as a randomization of the heart position (Fig. 4a). Abnormal right jogging (6.7%), no jogging (60.0%) and cardia bifida (6.8%) were observed along with normal left jogging (Fig. 4b). LR asymmetry in the liver, gut, and pancreas was also affected by *mcam* deficiency (Fig. 4c), with the following patterns observed: normal (54.7%), reversed (22.6%), bilateral (9.4%) and absent (13.2%; Fig. 4d). DFC injection of *mcam* mRNA can rescue the global defects of LR asymmetry in DFC^*mcam* MO^ embryos (Fig. 4b,d).

To explore whether the control of LR asymmetry by MCAM is conserved across vertebrates, we also examined Mcam expression and knockdown effects on organ laterality in *Xenopus laevis*. *Xmcam* mRNA was detected by WISH in the gastrocoel roof plate (GRP), the ciliated LR organizer of frogs. Injection of *Xmcam*-MO into cell lineages of the dorsal marginal zone of blastomeres, also shortened cilia length relative to control MO-injected embryos (Supplementary Fig. 11). The positions of the gall bladder, heart, and gut looping were inverted (situs inversus) or developed discordantly (heterotaxia) (Fig. 4e,f). These results suggest that MCAM plays conserved roles in LR organizer morphogenesis as well as in LR asymmetric patterning during vertebrate development.

### MCAM activates NFAT as an anchor of PLC-γ

**Figure 5:**
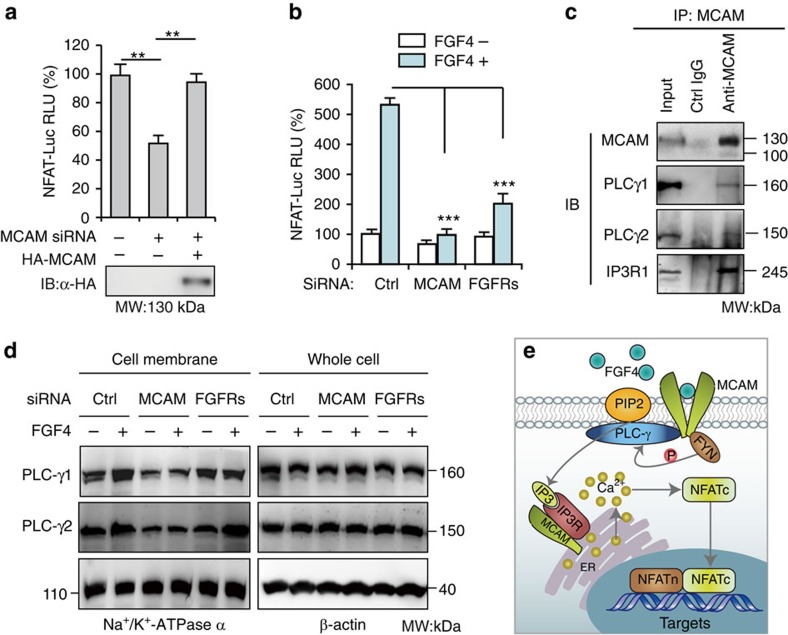
MCAM controls activation of NFAT. (**a**) NFAT-Luc reporter assay in HEK293 cells transfected together with MCAM RNAi or MCAM expression vector. RLU means relative luciferase activity. Data are presented as mean±s.e.m. (*n*=3). One-way ANOVA with Tukey's post-test and **P value<0.01. (**b**) After co-transfection of reporter plasmids and RNAi for 20 h, cells were cultured with serum-free medium overnight. NFAT-Luc activity was measured following 1 h treatment with FGF4 (2 ng/ml). No treatment served as negative control. Data are presented as mean±s.e.m. (*n*=3). Two-way ANOVA with Bonferroni post-test and ***P value<0.001. (**c**) Immunoprecipitation of MCAM followed by immunoblotting (IB). (**d**) PLC-γ abundance in membrane fraction of HEK293 cells and in whole cell lysate after a 48 h transfection with RNAi with or without treatment with FGF4 for 1 h. (**e**) Model of MCAM-controlled NFAT activation responding to FGF4. Once dimerization, MCAM conjugates FYN kinase to activate PLC-γ.

Next, we explored the molecular mechanisms underlying the roles of MCAM in lumenogenesis and ciliogenesis. To identify whether MCAM modulates the PLC-γ-initiated FGF signalling pathway, the NFAT reporter encoding consensus sequence recognized by a NFAT transcription factor was generated. MCAM knockdown drastically suppressed the activation of NFAT (by 45%; *P*=0.008) and these reductions were rescued by reconstitution of MCAM (Fig. 5a; Supplementary Fig. 12). There were no direct interactions between MCAM with FRS2 or growth factor receptor-bound protein (Grb) 2, which are critical intermediate signalosomes between activated FGFR and ERK–c-Fos–AP1 or PI3K–PDK–FOXO pathways. The activation of AP-1 or FOXO upon FGF4 stimulation was significantly inhibited in cells with FGFRs knockdown, compared with those cells with MCAM knockdown. In contrast, the effects of MCAM depletion on NFAT activation are higher than those of FGFR1–4 depletion (Fig. 5b; Supplementary Figs 13–15).

In addition, the NFAT upstream elements of PLC-γ1, PLC-γ2, and IP_3_R_1_ were also identified as candidate MCAM-interacting proteins in the yeast two-hybrid screen. These interactions were further confirmed by co-IP (Fig. 5c), suggesting that MCAM is a component of the IP_3_ receptor complex and works together with IP_3_R to promote intracellular Ca^2+^ release. Our findings thus consolidate the previous observations that MCAM resides in the endoplasmic reticulum (ER) and elevates intracellular Ca^2+^ levels at the MCAM-enriched regions^11^.

Recruitment of PLC-γ to the cell membrane is a prerequisite step for the catalysis of membrane-bound PIP_2_ as well as the subsequent Ca^2+^ release from ER^30^. We found that MCAM knockdown impaired PLC-γ1 and –γ2 anchoring to cell membranes in both the resting and activated status (that is, in the absence or presence of FGF4). By contrast, FGFR1–4 knockdown had no effects on PLC-γ recruitment onto cell membranes in either status (Fig. 5d,e). These results, together with previous findings that MCAM recruits Fyn kinase to activate PLC–γ^33^, not only demonstrate that MCAM can independently turn on PLC–γ, but also explain why the knockdown effects of MCAM on NFAT activation are higher than those of FGFRs.

### MCAM drives lumenogenesis through NFAT pathway

To evaluate the roles of NFAT activation on morphogenesis, we first blocked NFAT activity in the *in vitro* 3D cyst cultures with 1 μM of 11R-VIVIT (MAGPHPVIVITGPHEE), a specific peptide inhibitor of NFAT^34^. Statistical analysis indicated that NFAT blockage significantly disrupted lumen formation (Supplementary Fig. 16), which was similar to that of MCAM knockdown (Fig. 2a,b). Thus, these *in vitro* data indicate that NFAT is a critical member for AB polarity-driven lumenogenesis.

**Figure 6:**
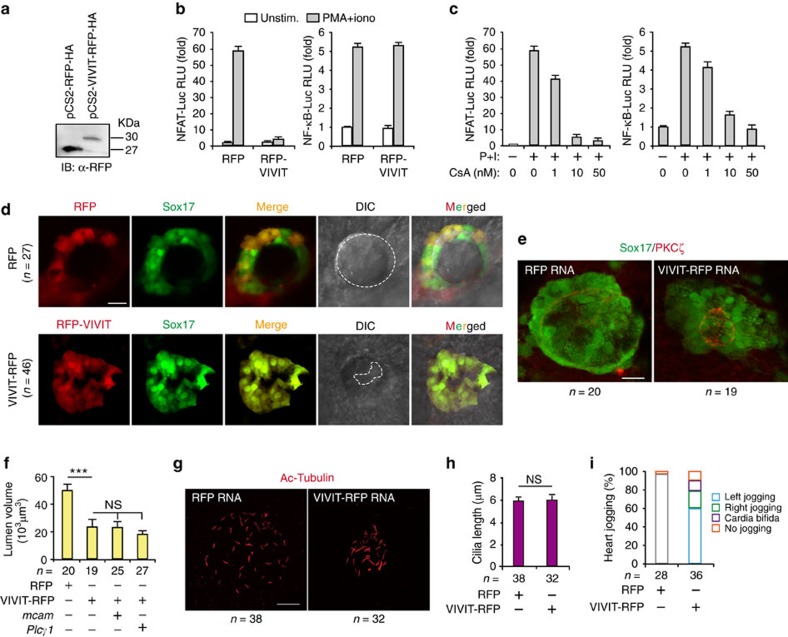
MCAM controls lumenogenesis by activation of NFAT. (**a**) Detecting the expression of RFP-HA and VIVIT-RFP-HA in HEK293 cells by IB. (**b**) VIVIT selectively inhibiting NFAT reporter activity. Jurkat cells were co-transfected with NFAT-Luc (left panel) or NF-kB-Luc (right panel) reporter plasmid, and with RFP and RFP-VIVIT expression plasmids. Twenty-four hours after transfection, cells were left untreated or were stimulated for 6 h with phorbol 12-myristate 13-acetate (PMA; 20 nM) and ionomycin (1 mM) (P+I). (**c**) Calcineurin dependence of NFAT and NF-kB reporter activity. Jurkat cells were transfected with NFAT-Luc (left panel) or NF-kB-Luc (right panel) reporter plasmid. Twenty-four hours after transfection, cells were left unstimulated or were stimulated for 6 h with P+I in the absence or presence of 1 μM cyclosporin (CsA). (**d**) Both RFP-tag and VIVIT-RFP were localized and expressed in DFC after microinjection of RFP and RFP-VIVIT mRNA into DFC of zebrafish KV. DIC means digital image of contrast. (**e**,**f**) Messenger RNAs were injected into zebrafish DFC of *Sox17:GFP* transgenic embryos, which were harvested at the 10 s stage. Lumen cells were labelled with an antibody against aPKCζ (red). Data are presented as mean±s.e.m. One-way ANOVA with Tukey's post-test. ***P value<0.001 and NS=not significant. (**g**,**h**) KV cilia were labelled with an antibody against acetylated tubulin after injection of the indicated mRNAs. Data are presented as mean±s.e.m. and analysed using unpaired student's *t*-test. The NS means not significant. (**i**) Quantitative analysis of heart joggings after injection of the indicated mRNAs into DFC of *Sox17:GFP* zebrafish embryos. Normal (left), reversed (right) and absent (no) jogging were calculated. Scale bar, 20 μm. n, number of observed embryos (**e**-**i**).

To further evaluate the *in vivo* roles of NFAT in lumenogenesis, we constructed a plasmid containing the VIVIT sequence. The expression of the red fluorescent protein (RFP)-tagged VIVIT blocked activation of an NFAT reporter but not of an NF-κB reporter in Jurkat cells stimulated with phorbol ester (PMA) plus ionomycin (Fig. 6a,b), although both reporters were equivalently sensitive to the inhibition of calcineurin with cyclosporine A (CsA; Fig. 6c).

Similar to observations in DFC^*mcam* MO^ embryos (Fig. 2), DFC injection of VIVIT mRNA caused about a 60% decrease in KV luminal volume in these zebrafish morphants, as compared to DFC^RFP mRNA^ embryos (5.3 × 10^4^ versus 2.1 × 10^4^ μm^3^; *P*<0.001; Fig. 6d,e). The volume reduction in zebrafish DFC^VIVIT mRNA^ embryos could not be rescued by injecting DFC with *mcam* or *PLC-γ1* mRNA (Fig. 6f). In contrast, blocking NFAT in DFC did not affect cilia length (Fig. 6g,h). Therefore, these *in vitro* and zebrafish *in vivo* data demonstrate that NFAT is the effective cascade downstream from the MCAM/PLC-γ pathway to fulfil lumenogenesis.

Consequentially, blocking NFAT in zebrafish DFC resulted in a severe disruption of cardiac jogging. Abnormal right jogging (20.1%, *n*=136), no jogging (10.3%, *n*=53) and cardia bifida (10.8%, *n*=30) were observed along with normal left jogging in zebrafish embryos (Fig. 6i). The high similarities in defects of luminal formation resulting from MCAM deficiency and NFAT depletion imply that MCAM may be the immediate upstream regulator of NFAT.

### MCAM constitutively activates JNK to determine ciliogenesis

**Figure 7:**
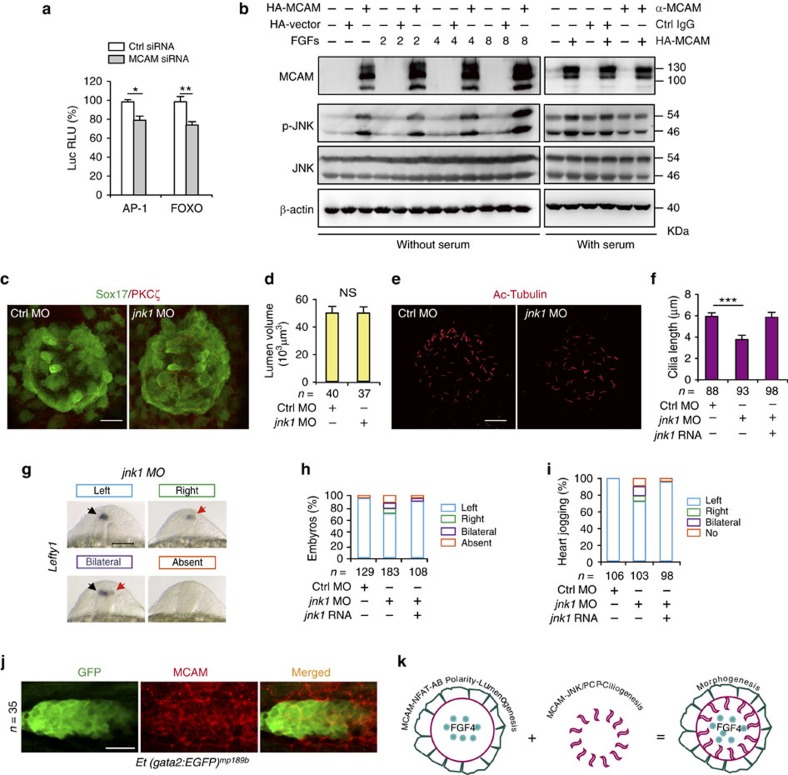
MCAM-dependent JNK activation regulates ciliogenesis. (**a**) Luciferase activity measured 30 h post transfection. AP-1-Luc or FOXO-Luc reporter vectors were co-transfected with either MCAM RNAi or control RNAi. Data (mean±s.e.m.) were analysed with unpaired student's *t*-test. *P value<0.05 and **P value<0.01. (**b**) Phosphorylation and expression of JNK following overexpression of MCAM by transfection with pCS2-MCAM-HA or blockage of MCAM by its functional antibody AA98. (**c**,**d**) DFC-specific knockdown of *jnk1* did not affect lumen formation in *Sox17:GFP* zebrafish embryos. Lumen cells were labelled with an antibody against aPKCζ (red). Scale bar, 20 μm. Data (mean±s.e.m.) were analysed with unpaired student's *t*-test. The NS means not significant. (**e**,**f**) DFC-specific knockdown of *jnk1* shortened KV cilia. The cilia were labelled with acetylated tubulin antibody. Scale bar, 20 μm. Data (mean±s.e.m.) were analysed using one-way ANOVA with Tukey's post-test. ***P value<0.001. (**g**,**h**) Randomized expression of left side-specific *lefty1* in the DFC^*jnk1* MO^ morphants. Scale bar, 100 μm. (**i**) Quantitative analysis of embryos with normal (left-sided), reversed (right-sided), cardia bifida (bilateral) and absent expression. n, number of embryos (**d**,**f**,**h**,**I**,**j**). (**j**) MCAM is localized at the zebrafish PLL system. Embryos of Et (gata2:EGFP) ^mp189b^ zebrafish line at the 28 h.p.f. stage were collected and stained with MCAM antibody. Scale bar, 15 μm. (**k**) A model of MCAM-mediated coordination of cell polarity during morphogenesis. See text for details.

Then we explored which signalling cascade downstream from MCAM is responsible for ciliogenesis. The MCAM knockdown inhibited reporter's activation of AP-1 (by 25%; *P*=0.045) and FOXO (by 30%; *P*=0.008; Fig. 7a). Subsequently, we examined the activation status of the key kinases upstream of these two transcriptional factors. We found that MCAM overexpression induced a robust increase in JNK activity with or without FGF2, 4 or 8 stimulation, which was suppressed by blocking MCAM with MCAM's functional antibody of AA98 (Fig. 7b). In contrast, overexpression or blockage of MCAM had no effects on the change of ERK, p38 and PDK phosphorylations. ERK phosphorylation was completely dependent on the presence of FGFs, but not on the abundance of MCAM (Supplementary Fig. 17). These results thus demonstrate that MCAM constitutively activates JNK, independent of FGF signals.

We next asked whether *jnk* is linked with Mcam-regulated KV morphogenesis or ciliogenesis. WISH data with zebrafish embryos indicated that like *mcam*, *jnk1* mRNA expression appeared to be ubiquitous, including in zebrafish KV (Supplementary Fig. 18). The KV co-expression of Mcam and JNK manifests the spatial rationality that Mcam can access and activate JNK during KV morphogenesis. Next, we found that the DFC knockdown of *jnk1* in zebrafish did not affect KV luminal volume (Fig. 7c,d). Notably, DFC knockdown of *jnk1* resulted in a reduction of KV cilia length by 17% relative to DFC^*control* MO^ zebrafish embryos (4.82 versus 5.85 μm; *P*<0.001). Such reduction was rescued by the DFC injection of *jnk1* mRNA into those morphants (Fig. 7e,f). The *jnk1* knockdown consistently induced randomized expression of *spaw* and its target genes, including *pitx2*, *lefty1* and *lefty2*, and caused the significant defect of LR asymmetry in zebrafish embryos (Fig. 7g–i; Supplementary Fig. 19). These results demonstrate that constitutive activation of JNK by MCAM is crucial for the determination of cilia length as well as LR asymmetry.

Polarized rosettes are common intermediates during morphogenesis of diverse organs, such as the zebrafish KV, the vertebrate pancreas, as well as the neural stem cell niche. We then asked whether or not the functions of Mcam in morphogenesis are only limited to KV rosette formation. Beyond KV, the zebrafish PLL system is another widely used model to investigate the cell polarity-driven morphogenesis. Next, we used the transgenic zebrafish Et (gata2:EGFP) ^mp189b^ line to trace the development of the PLL system^35^. Labelling endogenous Mcam with immunostaining in this line demonstrates that Mcam is also localized at the PLL system (Fig. 7j and Supplementary Fig. 20), suggesting that MCAM-mediated coordination of AB polarity and PCP is a broadly utilized mechanism during morphogenesis (Fig. 7k).

## Discussion

A major challenge in understanding morphogenesis is to decipher mechanisms underlying the coordination of AB polarity and PCP using genuine tissues. Here we reveal how MCAM simultaneously couples these two distinct patterning systems for lumen formation and cilium growth at the molecular, cellular and *in vivo* levels. Thus, this study sheds light on the mechanisms by which cell populations synchronously develop distinct morphologies during organ formations.

The unsolved central question in comprehending mechanisms of AB polarity is to decode the nature of *in vivo* polarity cues^1^,^36^. In this study, at both molecular and cellular levels, we demonstrate that FGF4 acts as a spatial cue for biogenesis of apical surface. Thus, in light of this study, morphogens with chemoattractant activity may have the potential to serve as *in vivo* spatial cues for the establishment of AB polarity. Furthermore, the cooperation of MCAM/FGF4 in AB polarity suggests that MCAM could be crucial in FGF4-executed morphogenetic events.

Another open question in cell polarity is to identify the critical polarity complexes that regulate polarized endocytosis^1^. The machinery of endocytic transportation is composed of secretory organelles, including the ER, Golgi complex and endosome. Our findings that MCAM interacts with IP_3_R_1_ of the ER, and mediates endocytic vesicular transportation strongly suggest that MCAM is a crucial player in the apical polarization of membrane proteins.

Although it has been well established that PIP_2_ plays a predominant role in the generation of intracellular asymmetry and an apical surface, the upstream signals initiating PIP_2_ asymmetry remain elusive^3^,^37^,^38^. Our findings that MCAM can independently anchor PLC–γ, which is also anchored onto the cellular membrane by its interaction with PIP_2_, suggest that MCAM, PLC–γ, and PIP_2_ could form a ternary complex. Therefore, such findings suggest the possibility that the apical distribution of MCAM on a cellular surface could contribute to the origination of PIP_2_ apical asymmetry.

Formation of cellular polarity requires the orchestration of intracellular signalling events^39^. Our study implies that the FGF4-MCAM-NFAT axis represents an essential signalling pathway for AB polarity as well as lumenogenesis. PLC-γ is a shared signalling nexus that acts downstream from more than 100 cell surface receptors^40^,^41^. In this regard, PLC-γ-mediated NFAT activation, elicited from signalling receptors other than MCAM, relies on, at least partially, MCAM-mediated PLC-γ activation. Thus, MCAM may be the long-sought-after upstream receptor of NFAT that integrates divergent intracellular Ca^2+^ signals with NFAT-dependent transcriptional response, for lumenogenesis.

It is well established that JNK activation is dependent on outside environmental signals. Thus, our finding that MCAM constitutively activates JNK is a novel scenario, which facilitates the understanding of MCAM as a requirement in JNK/PCP-dependent ciliogenesis^42^. Meanwhile, this constitutive activation of JNK by MCAM can also explain why there is increasing evidence linking MCAM to a wide spectrum of JNK-involved morphogenetic processes.

To our knowledge, our findings provide the first *in vivo* evidence on how two distinct patterning systems are integrated in genuine tissues and will facilitate better understanding of the key molecular and cellular players of morphogenesis. Therefore, this study is expected to shed light on the general mechanism by which cell populations coordinate two distinct cell polarities during morphogenesis.

## Methods

### Antibodies

AA1 (1:1,000) and AA98 (1:1,000), both of which are murine anti-MCAM monoclonal antibodies generated in our laboratory, recognize the first V domain and the last C2 domains in the extracellular region of MCAM, respectively. Additional information for all commercial antibodies is as following. Anti-FGF4 (1:1,000, orb39033) antibody was purchased from Biorbyt limited. Anti-HA-Tag (1:5,000, #3724), anti-Myc-Tag (1:5,000, #2276), anti-FGFR1 (1:1,000, #9740), anti-PLC-γ1 (1:1,000, #2822), anti-PLC-γ2 (1:1,000, #3872), anti-JNK (1:1,000, #9252S), anti-phospho-JNK (1:1,000, #9251S), anti-ERK (1:1,000, #9107), anti-phospho-ERK (1:1,000, #4370), anti-p38 (1:1,000, #9107), anti-phospho-p38 (1:1,000, #4511), anti-PDK (1:1,000, #3061), anti-phospho-PDK (1:1,000, #5662), Alexa Fluor 488 Phalloidin (1:2,000, 8878S) and the secondary antibodies of donkey anti-rabbit Alexa Fluor 555 (1:2,000, 8953S) antibodies were from Cell Signaling Technology. Anti-FGF3 (1:2,000, ab10830), anti-FGF8 (1:2,000, ab181492), anti-CDC42 (1:500, ab187643) and anti-PKCζ (1:2,000, ab119291) antibodies were from Abcam. Anti-FGFR2 (1:2,000, LS-C97522) and anti-IP3R1 (1:2,000, LS-C71062) were from Lifespan. Anti-PAR3 antibody (1:2,000, 07-330) was from Millipore. Anti-FGF2 (1:2,000, AJ1292A), anti-FGFR3 (1:2,000, AP14841c) and anti-FGFR4 antibodies (1:2,000, N-term, AP7639a-ev) were from Abgent. Anti-FRS2-α (1:200, A-5, sc-17841), anti-Grb2 (1:200, sc-255P), anti-Na^+^/K^+^-ATPase-α subunit (1:200, sc-21712), anti-Cdc42 (1:200, sc-87) and anti-β-actin (1:200, sc-8432) antibodies were from Santa Cruz Biotechnology. Anti-RFP-Tag (1:2,000, KM8082) was from Tianjin Sungene Biotech Co., Ltd. The donkey anti-mouse Alexa Fluor 488 (1:2,000, A11053) was from Invitrogen. The secondary antibody, HRP-conjugated goat anti-mouse (1:20,000, 31446) or rabbit IgG (1:20,000, 31431), was from Thermo Scientific Pierce.

### Reagents

Chemicals such as ionomycin, phorbol 12-myristate 13-acetate, cyclosporine, heparinase I, and heparinase III were from Sigma. All cell culture media were from Gibco and all transfection reagents were from Invitrogen. Protease inhibitor, Protein G PLUS-Agarose, and PhosSTOP (phosphatase inhibitor cocktail) were from Roche. The Matchmaker Gold Yeast Two-Hybrid System was purchased from Clontech. The Dual-Luciferase System (E1910) was from Promega. The membrane isolation kit was from Thermo Scientific. Heparan Sulfate Proteoglycan (HSPG, PA565Hu01) was from USCN life Science Inc. Human serum albumin recombinant was from California Bioscience. The recombinant FGF4 (AF-100-31) was from Peprotech, and FGF8a (4745-F8-050) was from R&D Systems. The recombinant human proteins of FGF2 (10014-HNAE), FGFR1/Fc (10616-H03H), FGFR2/Fc (10824-H03H), MCAM/Fc (10115-H02H), and IgG1 Fc (10702-HNAH) were from Sino Biological Inc.

### Cell lines with culture conditions and treatments

All of the cell lines were obtained from ATCC, and were authenticated by single nucleotide polymorphism testing and mycoplasma contamination testing. The HEK293 (human embryonic kidney 293, CRL-1573) cell line and the human liver cancer cell line HepG2 (HB-8065) were maintained in EMEM with 10% fetal bovine serum (FBS). The human malignant melanoma cell line A375 (CRL-1619) was maintained in DMEM with 10% FBS. The breast cancer cell line MDA-MB-231 (HTB-26) was maintained in EMEM with 10% FBS and 0.1 mM of nonessential amino acid (NEAA). The MCF7 (HTB-22) breast cancer cell line was maintained in EMEM with 5% FBS, 0.1 mM NEAA and 1 mM Sodium Pyruvate. The human colon cancer cell line HT-29 (HTB-38) was maintained in McCoy's 5a medium modified with 10% FBS. The Jurkat cells (TIB-152) were maintained in a RPMI-1640 medium.

The following are the primers for construction of MCAM-RFP: forward, 5′-gaa ttctgATGGGGCTTCCCAGGCTGGTCT-3′ and reverse, 5′-ggatcccgCTAATGCCTCAGATCGATGTAT-3′. EcoRI and BamHI were the selected enzymatic sites for cloning procedures. Treatment of sections with 0.006 IU ml^−l^ heparinase I and heparinase III for 2 h at 37 °C, followed by the addition of fresh enzyme for an additional 2 h, completely removed endogenous HSPGs^43^.

### Yeast two-hybrid screen

The Matchmaker Gold yeast two-hybrid system (Clontech) was used according to the manufacturers' instructions. The yeast strain Y2HGold, which expresses bait protein from the plasmids of pGBKT7-MCAM-1, -2 or -3, was used to screen the pre-made universal human (normalized) cDNA library (Clontech) in the Y187 yeast strain using a yeast two-hybrid method. Coding sequences of the human FGF2, FGF4, FGF8, PLC-γ1, PLC-γ2 or IP_3_R_1_ cDNAs were screened from the human cDNA library and confirmed by the sequencing method. The primers for construction of pGBKT7-MCAM-1 (AA, 24-322), pGBKT7-MCAM-2 (AA, 323-560) and pGBKT7-MCAM-3 (AA, 583-646) are listed as follows (lower cases indicate added adaptor sequences). MCAM-1 forward, 5′-gccgaattcATGGTGCCCGGAGAGGCTGAGC-3′; and MCAM-1 reverse, 5′-ggatcccCTACTGACATTCATAGCGCCCAC-3′. MCAM-2 forward, 5′-gccgaattcATGGGCCTGGACTTGGACACCA-3′; and MCAM-2 reverse, 5′-ggatcccCTAGCCCCGGCTCTCCGGCTCC-3′. MCAM-3 forward, 5′-gccgaattcATGAAGAAGGGCAAGCTGCCGT-3′; and MCAM-3 reverse, 5′-ggatcccCTAATGCCTCAGATCGATGTAT-3′.

### Surface plasmon resonance measurements

The binding kinetics between the soluble FGF2, FGF4 or FGF8 to MCAM, FGFR1 or FGFR2, were analysed at room temperature on a Biacore T100 machine (GE Healthcare) with CM5 chips (GE Healthcare) according to a published protocol as following^44^. The buffer of 1 × PBS with 0.05% Tween 20 was used for all measurements. For surface plasmon resonance (SPR) measurements, two chips were used. Recombinant human chimera proteins of MCAM/Fc, FGFR1/Fc, FGFR2/Fc or IgG1 Fc, were immobilized on the CM5 chip. IgG1 Fc and PBS with 0.05% Tween 20 were used either as the negative chimera-protein or the blank solvent control. SPR chips were treated using the same protocol; immobilization was conducted using *N*-hydroxysuccinimide and *N*-ethyl-*N*-(dimethylaminopropyl) carbodimide, and neutralization was carried out with 1.0 M ethanolamine. Upon completion of data collection after each cycle, the sensor surface was regenerated by washing with 10 mM glycine-HCl. A series of concentrations were used for the designed experiments. Kinetic and equilibrium parameter (*K*_D_) values were calculated by fitting the raw sensor gram with the 1:1 binding model using the Biacore T100 evaluation software.

### Co-immunoprecipitation and immunoblotting

The HEK293 cells were collected with 5 mM EDTA in PBS and lysed for 1 h at 4 °C in a lysis buffer (containing 10 mM Tris–HCl, 100 mM NaCl, 50 mM NaF, 2 mM EDTA, 0.5 mM sodium vanadate, 1% NP-40, a protease inhibitor cocktail and a phosphor-stop cocktail, pH 7.5). After removal of cell debris by centrifugation for 15 min at 14,000*g*, proteins (∼500 μg) were pre-cleaned using normal mouse IgG and Protein G PLUS-Agarose. The pre-cleaned supernatants were incubated overnight with anti-MCAM antibody AA1 at 4 °C with rotations. The reaction was further incubated with Protein G PLUS-Agarose for three hours at 4 °C. The immunoprecipitates were rinsed three times with a washing buffer (1 M NaCl, 1% NP-40, 50 mM Tris–HCl, pH 7.5) and analysed through immunoblotting (IB) with the desired antibody. The IB was carried out using SDS–polyacrylamide gel electrophoresis (SDS–PAGE), and polyvinylidene difluoride membranes were visualized by the ECL immunoblot detection system. All uncropped western blots can be found in Supplementary Fig. 21.

### Immunofluorescence staining and confocal microscopy

Cells were cultured on μ-Slide Ibidi chambers. After 24 h, cells were exposed to a FGF4 gradient with 10 ng ml^−1^ of source, ensuring that the final FGF4 gradient ranges from 0∼4 ng ml^−1^. Post 2 h of FGF4-gradient exposure, chemotaxing cells were washed and fixed with 4% paraformaldehyde in PBS for 25 min. Cells were then permeabilized by treating them with PBS/0.1% Triton X-100 for 5 min, and then blocked by incubation with 3% BSA in PBS for 1 h. Cells were then incubated with primary antibodies against MCAM (1:100), FGFR1 (1:50), aPKCζ (1:100), and PAR3 (1:100) in PBS, with 3% BSA overnight at 4 °C. Cells were washed three times with PBS/0.1% Tween 20 and then incubated for one hour with fluorescence-conjugated secondary antibody at a dilution of 1:300 at 37 °C. After 45 min, the cells were washed with PBS/0.1% Tween 20 and then counterstained with DAPI for DNA. A confocal laser scanning microscope (Olympus FLUOVIEW FV1000) with a mounted Olympus IX81 digital camera was used for image acquisition.

### Chemotaxis assay and live-cell imaging

Chemotaxis experiments were carried out in μ-Slide Ibidi chambers according to the manufacturer's instructions, with modifications as described earlier^45^. A HEK 293 cell suspension was diluted to 3 × 10^6^ cells per ml. Six microliter of cell suspension was applied onto one of the filling ports of the μ-Slide using a 20 μl pipettor, and 6 μl of air was aspirated from the opposite filling port. The slides were then placed in a sterile 10 cm Petri dish with a wet tissue around the slide and were transferred to a 37 °C incubator. After the cells attached, all plugs were gently removed from the filling ports and both reservoirs were filled with 45 μl of chemoattractant-free medium. For chemoattractant application, one of the filling ports was filled with 18 μl of FGF4 (10 ng ml^−1^) source solution by removing 18 μl of chemoattractant-free medium from the other port on the same side of the device. All the ports were then closed with plugs. Cell migration was recorded by mounting the μ-Slide onto the stage of an inverted microscope (Eclipse Ti, Nikon), which was fitted with a 37 °C incubator (5% CO_2_). Movies were taken using the CSU-X1 spinning disk confocal scanner (UltraVIEW VoX, Perkin-Elmer), a × 60 lens (Nikon), and the Volocity 6.0 software. The movies were taken every minute for RFP-MCAM, endogenous MCAM, or endogenous FGFR1.

### Growth of cysts in a three-dimensional matrigel

Cyst cultures in the matrigel were carried out according to an established protocol as following^38^. The matrigel was first thawed at 4 °C overnight, and then the dishes (Nest, with 15 mm diameter) were coated with 120 μl of Matrigel. The culture dishes were incubated for 15–30 min at 37 °C to allow the Matrigel to gel. A single cell suspension with 4 × 10^4^ cells per ml in 2% Matrigel was made. The 1,200 μl of cell suspension was plated onto the Matrigel-coated surface. The cultures were maintained for 5–7 days, replacing the Matrigel-medium mixture every two days until cysts with lumen formed. The nuclei of cysts were counterstained with DAPI (0.5 μg ml^−l^ in PBS) for 5 min at room temperature. Photos were taken with a confocal laser scanning microscope (Olympus FLUOVIEW FV1000) with an Olympus IX81 digital camera.

### Fish handling

The zebrafish lines of Tubingen strain, Tg (sox17: GFP) s870 and Et(gata2a:EGFP)mp189b 35 were maintained following standard protocols. Embryos were raised in an E3 buffer at 26–30 °C. All zebrafish experiments were conducted in an ethical manner as approved by the Animal Care and Use Committee of Tsinghua University. Embryo stages, according to the established criterion^46^, are indicated in the relevant figures and legends. WISH was performed according to standard procedures. For DFC^MO^ experiments, a volume of 1 nl fluorescent MO was injected into embryo yolks at the 500–1,000 cell stage, and embryos were selected by fluorescence microscopy for MO enrichment as the established standard^28^.

### RNA probes of zebrafish

Digoxigenin-labelled RNA probes of zebrafish *mcam*^24^, *fgf4*, *fgfr1*, *foxa3* (ref. 47), *cmlc2* (ref. 48), *southpaw*^49^, *pitx2* (ref. 50), *lefty1* (ref. 51) and *lefty2* (ref. 51) were synthesized by *in vitro* transcription using T7 or SP6 RNA polymerase.

### Antisense morpholino oligonucleotides

Morpholino oligonucleotides (MOs) were synthesized by Gene-Tools, LLC (Corvallis, OR). The sequences of MOs used in the zebrafish were as follows: *mcam* MO^24^, 5′-AGCAGTGCGGTGTAGGTCATTTCTC-3′; *mcam* MO 5′ mis control^24^, 5′-AGGCGTGCGGAGTAGCTCATTTGTC-3′; *fgf4* MO^52^, 5′-GCTACCGTTTTTCTCTATGCTTGAG-3′; *fgfr1* MO^17^, 5′-GCAGCAGCGTGGTCTTCATTATCAT-3′; *jnk1* MO (designed based on GenBank sequence AB030900), 5′-ACTGTATCCTGGGCATTCAAGAAG-3′; *jnk1* MO 5′ mis control, 5′-ACTGTATGCTCCCGATTGAAGAAG-3′. The MO of Z-*mcam* was injected with a dosage of 4 ng for each embryo.

Sequences of morpholinos used in *Xenopus laevis* were as follows: *Xmcam*-MO, 5′**-**ATGAGAGAAGTCATGGTCCTGCTAC-3′; *Xmcam* MO 5′ mis, 5′-ATCAGAGAACTCGTGATCCTGCTTC-3′. The MO of *Xmcam* was injected with 15 ng for each embryo.

### Kupffer's vesicle flow analysis

Zebrafish embryos at the 6 to 8 somite stage were dechorionated and mounted in a 1% low melt agarose. Fluorescent beads (0.5–2 μm, Polyscience, Inc) were injected into the KVs and imaged on a Leica DMRA compound microscope, using a camera (UltraVIEW VoX, Perkin-Elmer) with a × 40 lens. Metamorph software (Universal Imaging Corp.) was used to track the injected beads and to calculate bead velocity.

### *Xenopus laevis* handling and RNA probe of *Xenopus laevis*

Xenopus laevis frogs (Nasco, Atlanta, GA, USA) were stimulated to lay egg by injection of human chorionic gonadotropin. Embryos were obtained by *in vitro* fertilized and dejellied with 3.2 μM DL-Dithiothreito, pH 8.9, and cultured in 0.1 × 0.1 × Marc's modified Ringer's (MMR; 0.1 M NaCl, 20 mM KCl, 1 mM MgSO_4_, 2 mM CaCl_2_, 5 mM Hepes, pH 7.8, 0.1 mM EDTA pH8.0)^53^. All animal work received ethics approval from the Animal Care and Use Committee of Institute of Biophysics, Chinese Academy of Sciences.

For Classification of organ situs defects, embryos at stage 45 were anaesthetized in 0.15% MS222 (Sigma-Aldrich, St Louis, MO, USA), 0.1 × MMR and recorded under stereo microscope (Olympus SZX16, Japan). Situs defects were classified into three types, complete inversion of organ situs (situs inversus), random organ transposition (heterotaxia) and situs solitus (wild type) according to deviation from the normal position and morphology^54^.

Probes for *Xenopus laevis mcam* and *Xnr-1* (ref. 55) were synthesized with linearized pCS2+HA-Xmcam (a full-length cDNA obtained as described below) and pCS2-Xnr-1 (a 388 bp fragment of the 5′-coding region) as templates, respectively.

### Synthesis of mRNAs

*In vitro* synthesis of mRNAs was performed using the mMessage mMachine kit (Ambion) from the following linearized pCS2+HA plasmids: Z-*mcam* forward, 5′-accatgggcATGACCTACACCGCACTGCT-3′; and Z-*mcam* reverse, 5′-ggatccTTCAGTGGGTGACTTTTTG-3′. Z-*fgf4* forward, 5′-accatgggcATGAGTGTCCAGTCGGCCCTC-3′; and Z-*fgf4* reverse, 5′-ggatccAATTCTAGGCAAGAAATGT-3′. Z-*fgfr1* forward, 5′-accatgggcATGATAATGAAGACCACGCTG-3′; and Z-*fgfr1* reverse, 5′-ggatccGCGCTTTTTAAAGGCCACTCCTC-3′. Z-*plcγ1* forward, 5′-accatgggcATGGCTGCGAGCGCGGGC-3′; and Z- *plcγ1* reverse, 5′-ggatccCGCTCGGTTATCGGCGCGAC-3′. Z-*jnk1* forward, 5′-accatgggcAGAAACTCAGCCGACCCTT-3′; and Z-*jnk1* reverse, 5′-ggatccGCAGCCAATAGACCAGACAT-3′. *Xmcam* forward, 5′-accatgggcATGAATTCTCTCATACTGCTT-3′; and *Xmcam* reverse, 5′-ggatccTCAGTTTCTTAAATCCATGTA-3′ (gene bank accession number KJ913668.1).

The coding sequence of the RFP-tagged NFAT inhibitor, RFP-VIVIT peptide (MAGPHPVIVITGPHEE), was inserted into the plasmid of pCS2+HA. The linearized pCS2+HA-VIVIT-RFP plasmid was used as the template for *in vitro* synthesis of the RFP-VIVIT mRNA. VIVIT forward 5′-accatgggcATGGCTGGGCCTCATCCCGTC-3′ and VIVIT reverse 5′-ggatccGGCGCCGGTGGAGTGACGGCCC-3′. The mRNA was injected into zebrafish or *Xenopus laevis* with a 250 pg dose of mRNA for each embryo.

### Gene silencing with siRNA and shRNA

SiRNAs were purchased from GenePharma. The siRNAs against FGFR1, FGFR2, FGFR3 and FGFR4 were designed to target their coding regions. The siRNAs against MCAM were designed to target at its 3′-untranslated region in such way that it results in the specific inhibition of endogenous MCAM RNA, without affecting exogenous transcripts. The concentration of each siRNA is indicated in the respective figures, and the transfection followed the manufacturer's instructions of Lipofectamine RNAi MAX. The siRNA sequences used were as follows: MCAM -#1 sense is 5′-CAGGUGAAUUAGCCUCAAUTT-3′. MCAM-#2 sense is 5′-GGUGUGUAAAUUUGCAAAUTT-3′. FGFR1-#1 sense is 5′-GGAGGUGCUUCACUUAAGATT-3′. FGFR1-#2 is SMART pool (M-003131-02) from Dharmacon (Chicago, IL). FGFR2-#1 sense is 5′-GGCCUCUCUAUGUCAUAGUTT-3′. FGFR2-#2 sense is 5′-GGACUUGGUGUCAUGCACCTT-3′.FGFR3-#1 sense is 5′-GCACACACGACCUGUACAUTT-3′. FGFR3-#2 sense is 5′-CGACAAGGAGCTAGAGGTT-3′. FGFR4-#1 sense is 5′-GCCGACACAAGAACAUCAUTT-3. FGFR4-#2 sense is 5′-CCACCACAUUGACUACUAUTT-3′. The plasmid of pGPU6/Neo-MCAM-shRNA was constructed by inserting the antisense sequence of the MCAM-siRNA-#1.

### Luciferase reporter assay

Cells (1 × 10^4^) were transfected with 0.2 μg of reporter constructs and 4 ng of pRL-TK (Promega) using Lipofectamine 2,000 reagent (Invitrogen). Cells were transfected with MCAM-siRNA or FGFR1–4-siRNAs, or co-transfected with MCAM-siRNA and HA-tagged MCAM-containing vectors for overexpression of exogenous MCAM. After 24 h of transfection, cells were treated with or without FGFs as indicated in the respective figures. Both firefly and *Renilla* luciferase activities were measured using a dual-luciferase reporter assay system in a Glomax multi-detection system luminometer (Promega). Firefly luciferase activity was normalized against *Renilla* luciferase activity, which was detected by co-transfection with pRL-TK in all reporter experiments. The luciferase activity was normalized against the luciferase activity of cells transfected with the pGL3-E control vector. The relative luciferase activities were expressed as fold increase over the paired control cells. The pGL3-E-NFAT, AP-1, FOXO or NF-κB plasmids were constructed by inserting consensus sequences to the respective transcription factor binding sites, and are indicated as following: pGL3-E-NFAT^56^, 5′-AggAAAAAC-3′; pGL3-E-AP-1 (ref. 57), 5′-ATgAgTCAT-3′; pGL3-E-FOXO^58^, 5′-gTAAACA-3′; pGL3-E-NF-κB^59^, 5′-gggACTTTC-3′.

### Zebrafish immuostaining

The embryos were collected at desired stages and their chorions were removed. They were then fixed with 4% paraformaldehyde overnight at 4 °C. The fixed embryos were dehydrated with sequentially graded PBST/methanol series (3:1, 1:1 and 1:3) and 100% methanol. They were stored at −20 °C for more than 30 min. After that, the embryos were rehydrated with sequentially graded methanol/PBST series (3:1, 1:1 and 1:3) and 100% PBST. The PBST was removed with 1 mM EDTA (pH 8.0) and the embryos were boiled in this EDTA solution for 10 min. They were then cooled slowly to room temperature. The embryos were washed with PBST and PBS-Triton X-100 (0.5%) sequentially. The embryos were blocked with a 3% BSA-PBST solution for at least an hour, and then incubated with anti-MCAM (1:100) overnight at 4 °C. After washing them three times with PBST, the embryos were incubated with the fluorescence labelled secondary antibody (1:200) overnight at 4 °C. The nuclei were stained with DAPI (1 μg ml^−l^). After washing three times with PBST, the embryos were mounted with coverslips and photographed with a confocal microscope.

### Statistical analysis

Statistical analysis was performed in SPSS. Data are shown as mean±s.e.m. All data sets were tested for normal distribution with normality tests before proceeding with parametric or non-parametric analysis. Unpaired Student's *t*-test was applied to data sets with normal distributions. When appropriate in case of multiple comparisons, one-way analysis of variance (ANOVA) with Tukey post-test or two-way ANOVA with Bonferroni post-test was applied. Statistical evaluation of experiments represented by bar graphs was performed using Pearson's chi-square tests. A value of *P*<0.05 was considered significant. In experiments of cell confocal microscopy, at least 100 samples were acquired to ensure adequate statistical power. For live-cell imaging experiments, embryo lumenogenesis analysis, and embryo organ patterning, the number of samples was mainly constrained by the complexity of the experimental procedure, data acquisition and analytical capacity. No exclusion criteria were used in experiments. To prevent selection bias, samples of cells and genotype matched pools were randomly divided into experimental groups. Data were analysed automatically where possible to avoid subjective assessments.

### Data availability

The DNA sequence of Xenopus *mcam* has been deposited inGene Bank under the accession code KJ913668.1. The authors declare that all the data supporting the findings of this study are available within the article and its supplementary information files or from the corresponding author upon reasonable request.

## Additional information

**How to cite this article:** Gao, Q. *et al*. The signalling receptor MCAM coordinates apical-basal polarity and planar cell polarity during morphogenesis. *Nat. Commun.* **8**, 15279 doi: 10.1038/ncomms15279 (2017).

**Publisher's note:** Springer Nature remains neutral with regard to jurisdictional claims in published maps and institutional affiliations.

## Supplementary Material

Supplementary Information Supplementary Figures.

Supplementary Movie 1 Temporal and spatial dynamics of endogenous MCAM responding to a FGF4 gradient.

Supplementary Movie 2 Temporal and spatial dynamics of RFP-Tagged MCAM responding to a FGF4 gradient.

Supplementary Movie 3 Temporal and spatial dynamics of endogenous FGFR1 responding to a FGF4 gradient.

Supplementary Movie 4 Movement of beads injected into the KV of control morphants.

Supplementary Movie 5 Movement of beads injected into the KV of mcam morphants.
