# Supplementary material for: The signalling receptor MCAM coordinates apical-basal polarity and planar cell polarity during morphogenesis
Source: Nat Commun. 2017 Jun 7;8:15279. doi: 10.1038/ncomms15279 (PMC5467231; doi:10.1038/ncomms15279)
Supplement: Supplementary Information — Supplementary Figures. [file ncomms15279-s1.pdf]

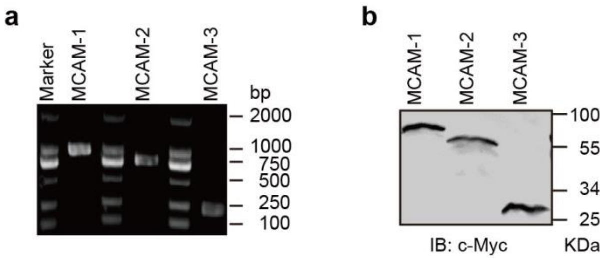

Supplementary Figure 1. Identifying the construction and expression of the truncated bait proteins of MCAM. (**a**) Three truncated constructs of pGBKT7-MCAM-BD (DNA-binding domain) 1–3 were identified by PCR. (**b**) Expression of c-Myc-fusion truncated versions of the bait proteins MCAM-1, MCAM-2, and MCAM-3 in Y2HGold yeast strain. Anti-c-Myc antibody was used for immunoblotting.

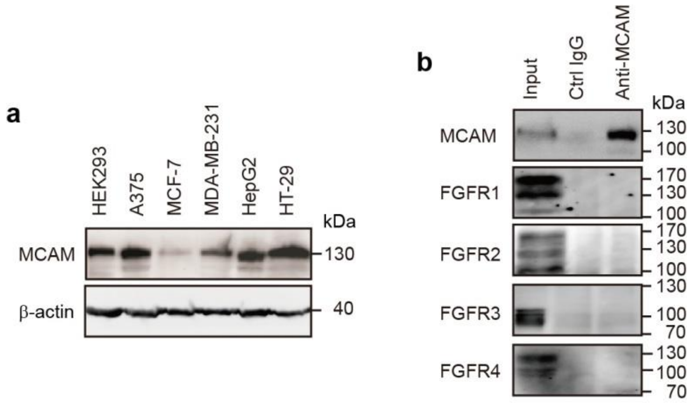

Supplementary Figure 2. Lack of interaction between MCAM and FGF receptors. (**a**) Endogenous MCAM protein levels were examined by immunoblotting using anti-MCAM antibody of AA1. (**b**) HEK293 cells were lysed and whole cell lysates were incubated with anti-MCAM antibody AA1, normal IgG as non-relevant antibody control. Fifty μg of whole cell lysate were loaded as input for positive control of immunoblotting. MCAM and FGF receptors of FGFR1 to FGFR4 were determined by immunoblotting using either anti-MCAM antibody AA1 (upper panel) or antibodies against FGF receptors (lower panels).

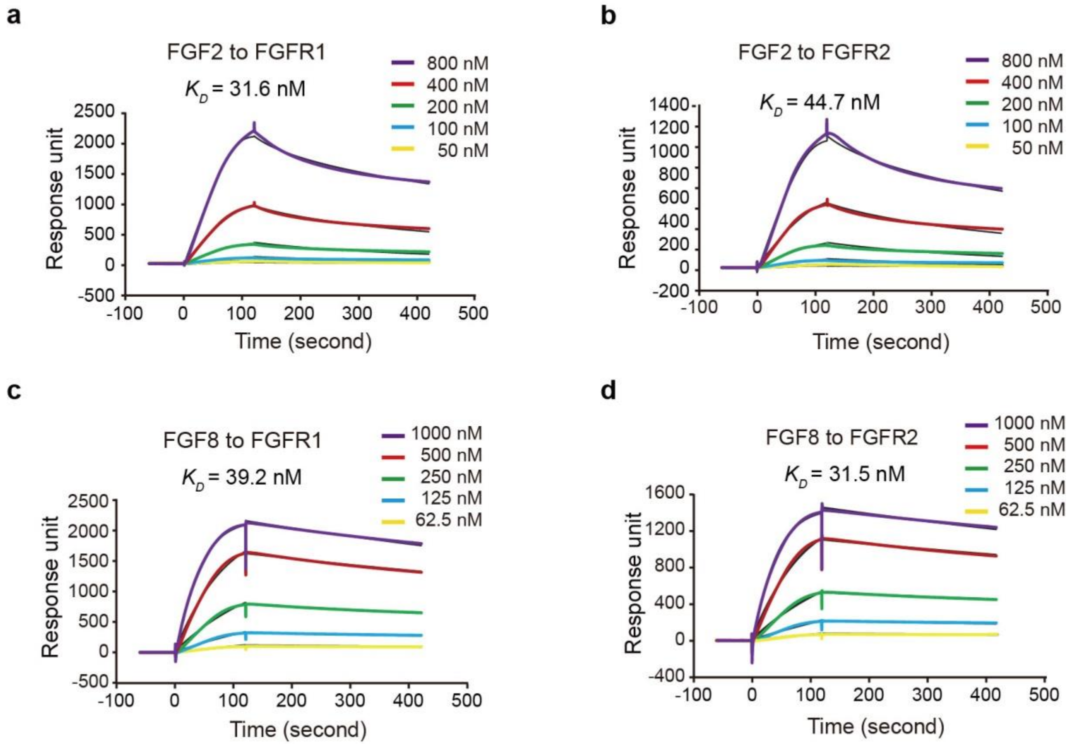

Supplementary Figure 3 Binding affinities of FGF2 or 8 to FGFR1 or FGFR2 analyzed by surface plasmon resonance. A gradient of concentrations of FGF2 and 8 were injected through flow cells immobilized with either FGFR1 or FGFR2. (**a, b**) The kinetic dissociation constants (K_D) of FGF2 binding to their receptors of FGFR1 (**a**) and FGFR2 (**b**) were calculated. (**c, d**) The kinetic dissociation constants (K_D) of FGF8 binding to their receptors of FGFR1 (**c**) and FGFR2 (**d**) were calculated.

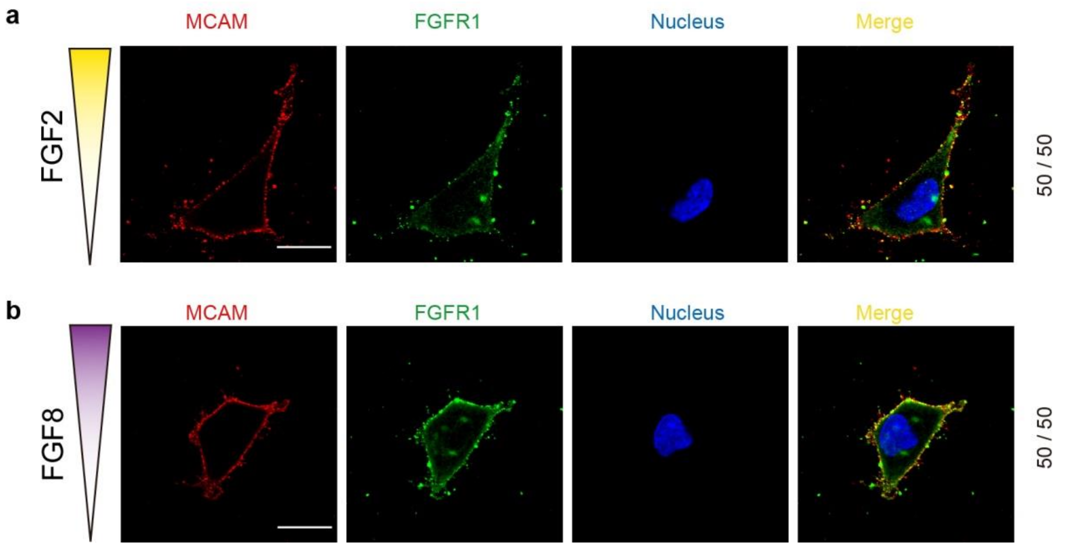

Supplementary Figure 4 Distribution of MCAM and FGFR1 along the gradient of FGF2 or 8. Immuno-staining of MCAM and FGFR1 in chemotaxing cells to an FGF2 (**a**) or 8 (**b**) gradients. The source concentration of FGF2 or 8 was 10 ng/mL. Scale bars, 20 μm.

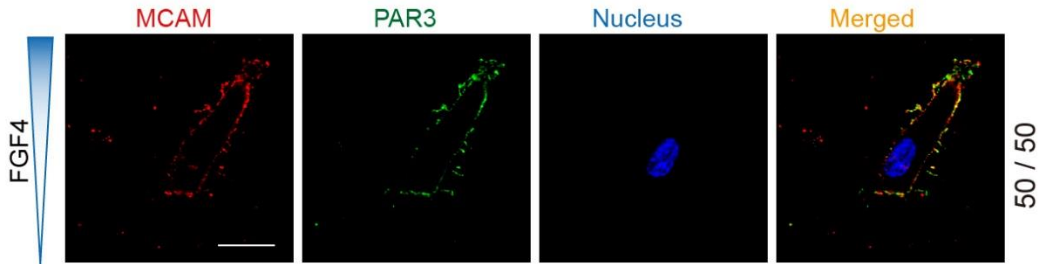

Supplementary Figure 5. Distribution of MCAM and PAR3 along the gradient of FGF4. Immuno-staining of MCAM and PAR3 in chemotaxing cells to an FGF4 gradients. The source concentration of FGF4 was 10 ng/mL. Scale bar, 20 µm.

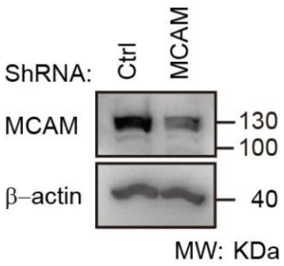

Supplementary Figure 6. MCAM-directed shRNA down-regulates its endogenous protein levels. Endogenous MCAM protein levels of HEK293 cells transfected with MCAM-shRNA were determined by immunoblotting using anti-MCAM antibody AA1, equal loading was estimated by β-actin expression.

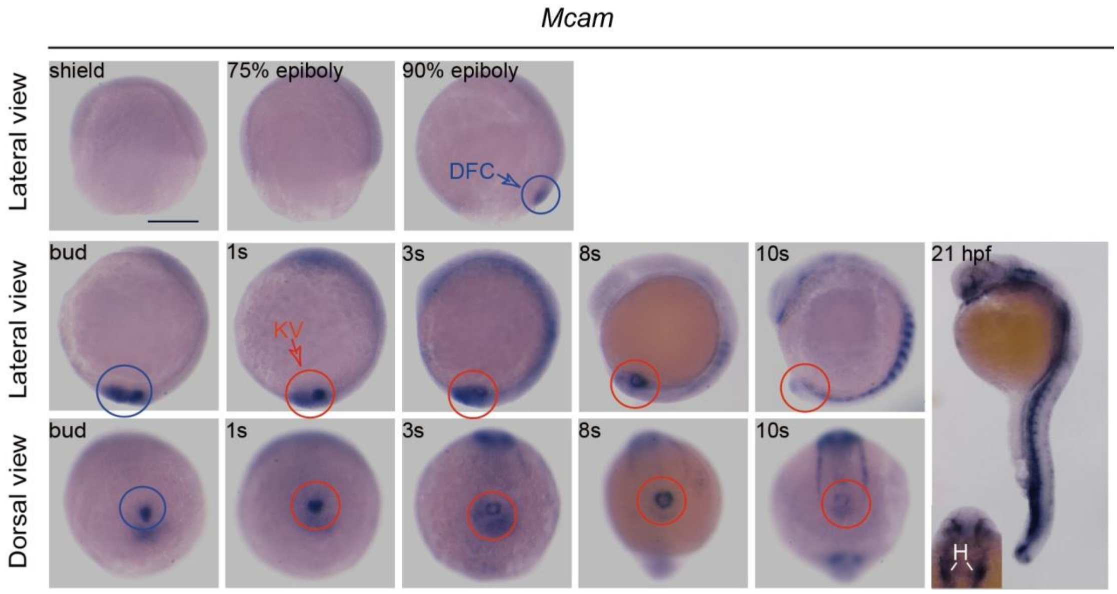

Supplementary Figure 7. The spatiotemporal distribution of *mcam* mRNA in zebrafish embryos. Zebrafish *mcam* mRNA expression was measured in DFCs (blue circles) and in KV (red circles) of wild-type embryos. At 21 hour post fertilization (hpf), *mcam* was expressed at blood vasculature, heart, somite, and eye field. The inset indicates expression of *mcam* mRNA in the heart. Inset: H, heart. Scale bar, 250 μm.

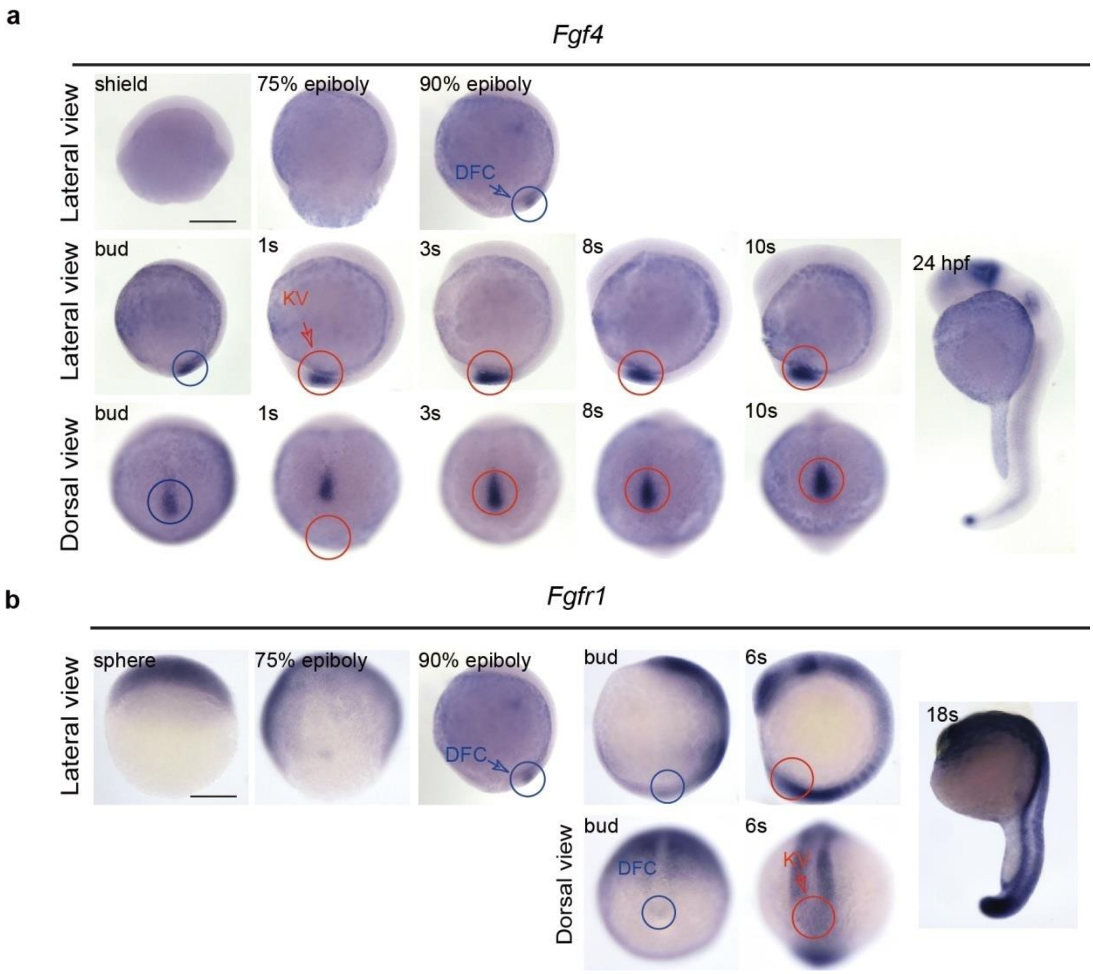

Supplementary Figure 8. The spatiotemporal distribution of the mRNA of *fgf4* and *fgfr1* in zebrafish embryos. (**a**) *Fgf4* was predominantly expressed in DFCs (blue circles), KV (red circles) and its peripheral region. *Fgf4* was also detected at the posterior notochord. At 24 hpf, *fgf4* was expressed in the midbrain–hindbrain boundary, pharyngeal arch and tail bud. (**b**) Weak expression of *fgfr1* RNA was detected in DFCs (blue circles) and in KV (red circles) of wild-type embryos. At 6s stage, *fgfr1* was also expressed in mesoderm, somite, eye field and hindbrain. The mRNA was detected in wild-type embryos by WISH. Lateral views show dorsal to the right and the head from the top; dorsal views show the head from the top. s, somite. Scale bar, 250 µm.

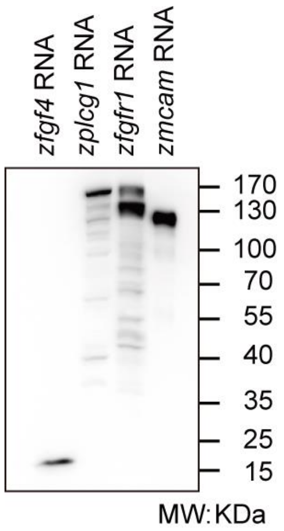

Supplementary Figure 9. Detecting the exogenous expression of zebrafish proteins. The zebrafish sequences of FGF4, PLCγ1, FGFR1, or MCAM were inserted into the HA-tagged pCS2+ plasmid. Their expression was detected using immunoblotting, in which the anti-HA-Tag antibody was used.

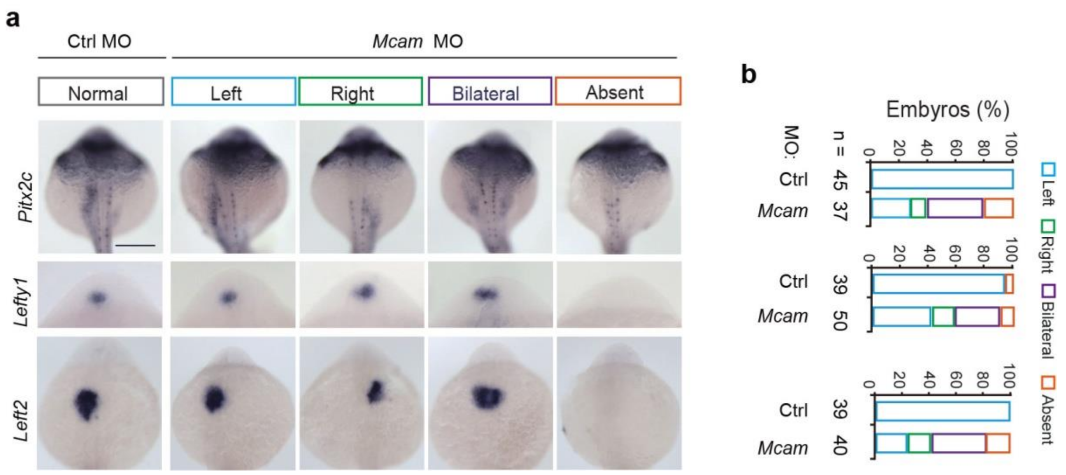

Supplementary Figure 10. MCAM dictates the expression of left side-specific genes in correct localization. (**a**) Randomized expression of left side-specific target genes of *pitx2* and *lefty1* and *2* in *mcam* morphants. Scale bar, 250 µm. (**b**) Quantitative analysis of embryos with normal (left-sided), reversed (right-sided), bilateral, and absent expression.

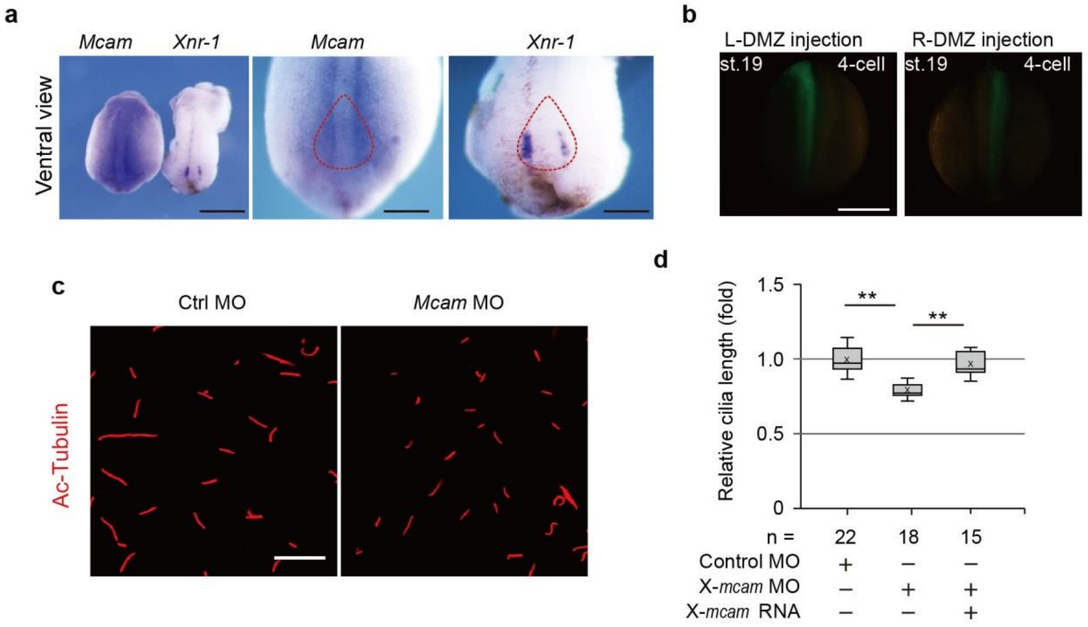

Supplementary Figure 11. Ciliary morphogenesis defects in gastrocoel roof plate of *Xenopus laevis* caused by X-*mcam* knockdown. (**a**) X-*mcam* locates at the boundary of the gastrocoel roof plate (GRP) in stage 17 embryos, similar to the location of *Xnr-1* (a marker probe of GRP). Scale bars: left, 1 mm; middle and right, 300 μm. (**b**) The stage 19 embryos microinjected with X-*mcam* MO and GFP mRNA into the left (L) or right (R) dorsal-marginal zone (DMZ) at 4-cell stage. Scale bar, 500 μm. (**c**) GRP cilia labeled with an antibody against acetylated tubulin after injection of embryos with X-*mcam* MO. Scale bar, 10 μm. (**d**) Quantification of relative cilia length of GRP cilia in defined areas, indicated by white squares. ''x'' in box plots represents mean values. Numbers indicate number of embryos or dorsal explants analyzed. Data are expressed as mean±s.e.m.; One-way ANOVA, Tukey's post-test; ***P* value <0.01.

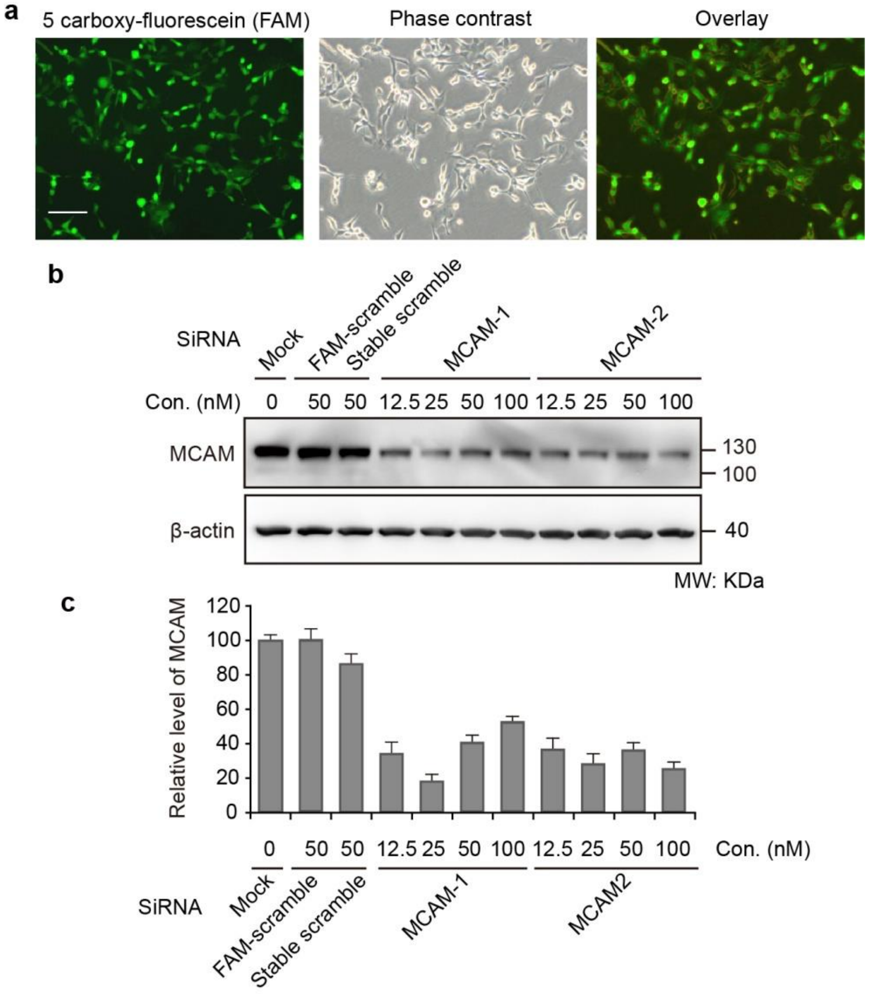

Supplementary Figure 12 Down-regulation of endogenous MCAM protein levels by MCAM siRNA. (**a**) SiRNA transfection efficiency was measured by tracing the presence of intracellular FAM. (**b**) Endogenous MCAM protein levels of HEK293 cells transfected with siRNA against MCAM were determined by immunoblotting using anti-MCAM antibody AA1, β-actin was used as equal loading control. Scrambled siRNA-FAM (5-carboxy-fluorescein) and Stable^{TM} (chemical modified) siRNA were used as controls. (**c**) The band density (mean ± s.e.m.) was measured from at least 3 independent immunoblots and was normalized to that of β-actin. The signal from cells transfected with scrambled siRNA was set to one. Scale bars, 200 μm.

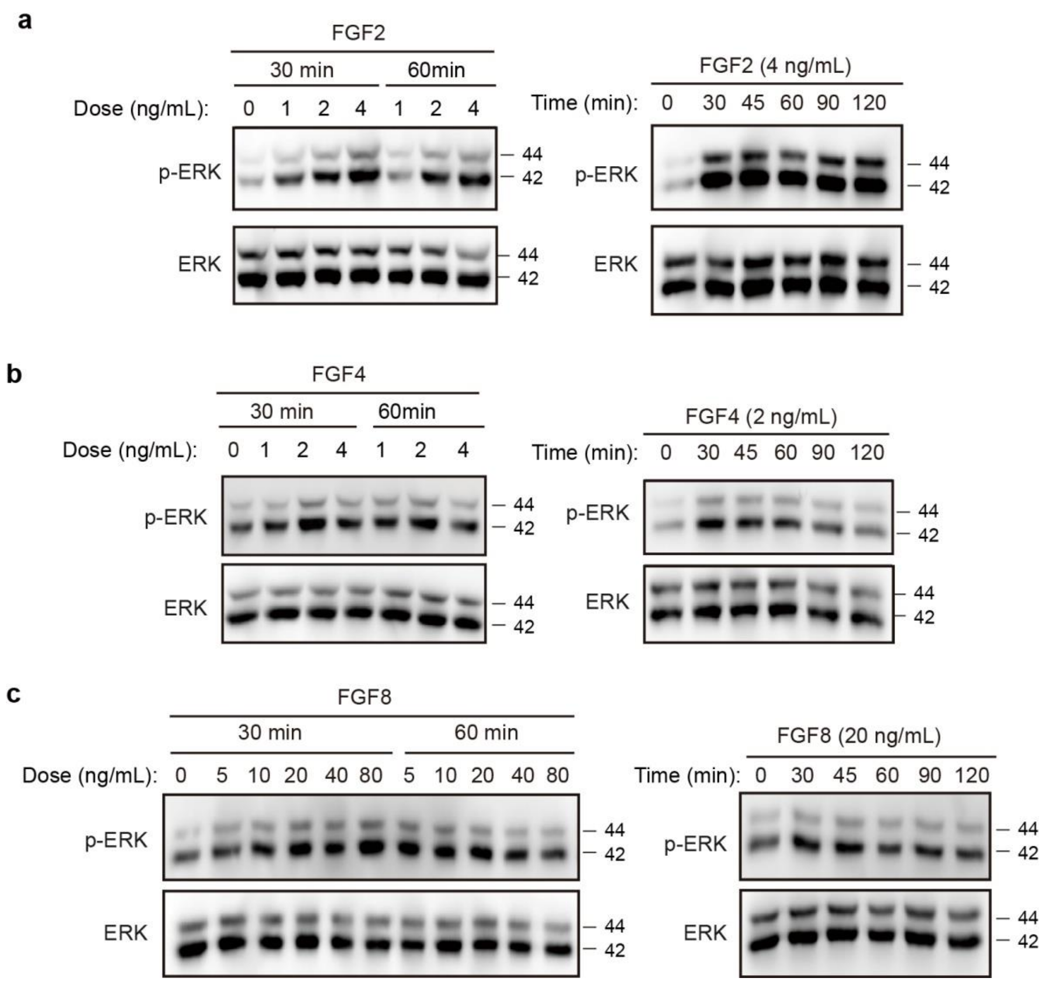

Supplementary Figure 13. Dose and time effects of FGF stimulation on ERK activation. HEK293 cells were starved through serum depletion for 24 h, and then incubated with FGF2 (**a**), 4 (**b**), or 8 (**c**) for the times and dosages indicated. ERK phosphorylation (upper panel) and total ERK protein levels (lower panel) were determined by immunoblotting using whole cell lysate.

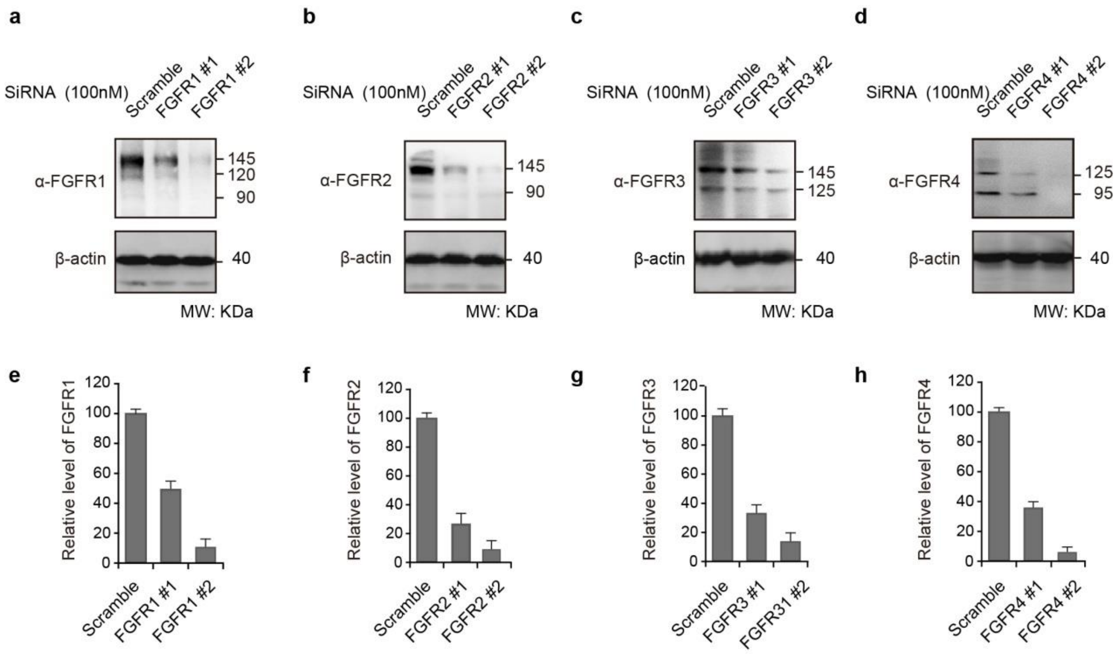

Supplementary Figure 14 Down-regulation of endogenous FGFRs protein levels by siRNA. (**a-d**) Endogenous levels of FGFR1-4 proteins in HEK293 cells transfected with respective siRNA against FGFR1-4 were determined by immunoblotting using anti-FGFR1-4 antibodies. β-actin was used as equal loading control. Scrambled siRNA was used as non-targeting and negative control. (**e-h**) The band density (mean ± s.e.m.) of FGFR1-4 was measured from at least 3 independent immunoblots and was normalized to that of β-actin. The signal from cells transfected with scrambled siRNA was set to one.

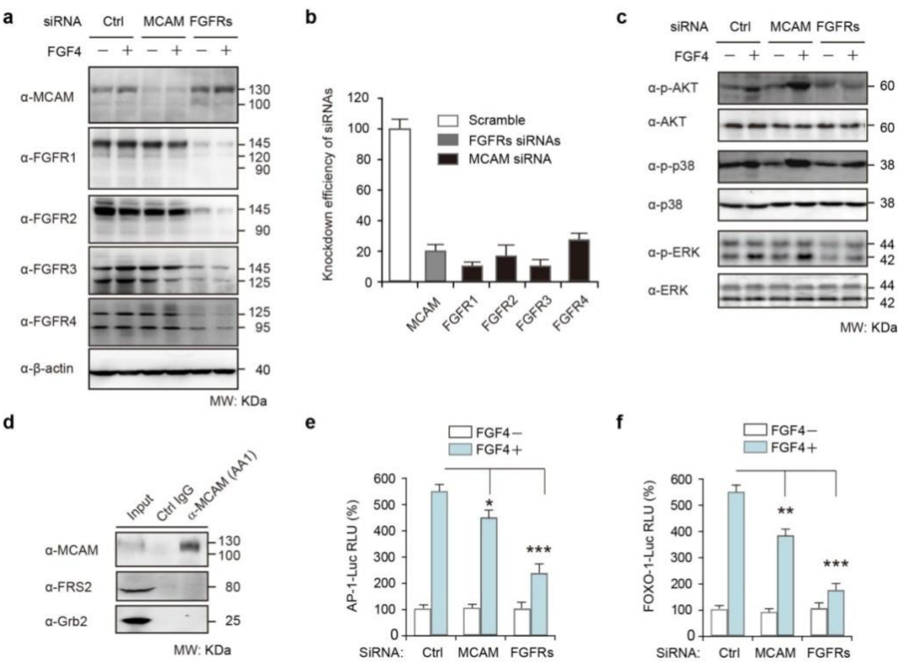

Supplementary Figure 15. Comparisons between MCAM and FGF functions on downstream signaling outputs responding to FGF4. (**a**) Endogenous proteins levels of MCAM or FGFR1-4 in HEK293 cells transfected with MCAM siRNA or siRNA mixture of FGFRs, were determined by immunoblotting using anti-MCAM or anti-FGFR1-4 antibodies. (**b**) The band density (mean ± SEM) was measured from at least 3 independent immunoblots and was normalized to that of β-actin. The signal from cells transfected with scrambled siRNA was set to one. (**c**) Examination of the phosphorylation and expression of AKT, p38, and ERK when cells were treated as in **a**. The concentration of FGF4 was used as 2 ng/mL. (**d**) No interaction between MCAM and the substrates of FGFRs, FRS2-α and Grb2. Immunoprecipitation of MCAM followed by immunoblotting of FRS2-α and Grb2. (**e, f**) After co-transfection of reporter and RNAi plasmids for 20 h, cells were cultured with serum-free medium overnight. AP-1-Luc or FOXO-Luc activity was measured following 1 h treatment with FGF4 (2 ng/mL). No treatment served as negative control. Data are expressed as mean±s.e.m.; two-way ANOVA, Bonferroni post-test; *P value<0.05, **P value<0.01, ***P value<0.001.

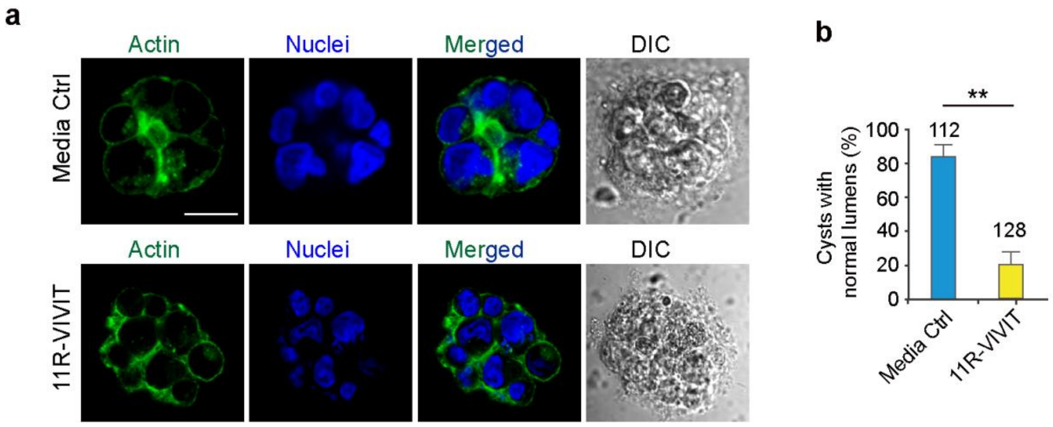

Supplementary Figure 16. The requirement of NFAT in proper luminal formation. (**a**) HEK293 cells were cultured on Matrigel for 5 days to enable cyst formation in the presence or absence of the NFAT inhibitor 11R-VIVIT (1 μM). Cyst nuclei and F-actin were labeled with DAPI and phalloidin, respectively. Scale bar, 20 μm. (**b**) Quantitative analysis of cysts with or without normal lumens following treatment with 11R-VIVIT or media (control). Percentage of cyst with normal lumens are plotted as mean±s.e.m.; control (blue bar) and 11R-VIVIT treatment (yellow bar) (n＝3). Pearson's chi-square tests and $^{**}P$ value < 0.01.

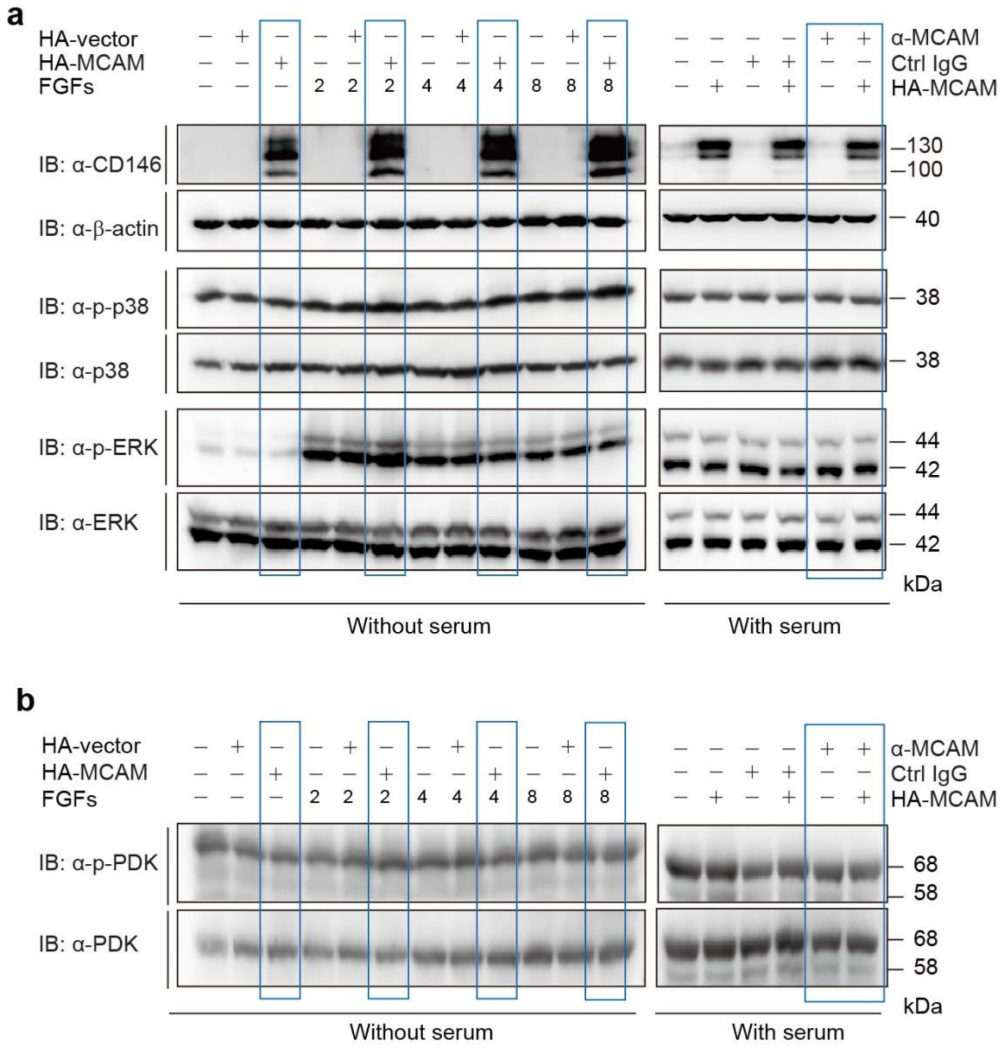

Supplementary Figure 17. The effects of manipulating MCAM expression levels on activation of MAPKs or AKT pathways. (**a**) Examination of the phosphorylation and expression of p38 and ERK following either MCAM over-expression by exogenous transfection with pCS2-MCAM-HA vector or MCAM blocking by its functional antibody AA98. (**b**) Phosphorylation and expression of PDK when cells were treated as in **a**. FGFs were used as the following concentrations: FGF2, 4 ng/mL; FGF4, 2 ng/mL; FGF8, 20 ng/mL.

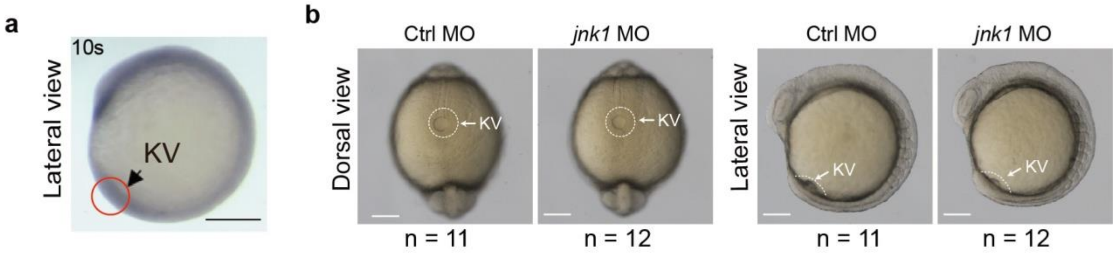

Supplementary Figure 18. *Jnk1* depletion had no effects on KV lumen formation. **(a)** *Jnk1* mRNA was detected in KV of wild-type embryos by WISH. Scale bar, 250 μm. (**b**) Embryos injected with *jnk1* or control MO at the 1-4-cell stage. Embryos were harvested at the 10s stage. The KV is delineated by circles or semi-circles in light micrographs of the live embryos. Scale bar, 300 μm.

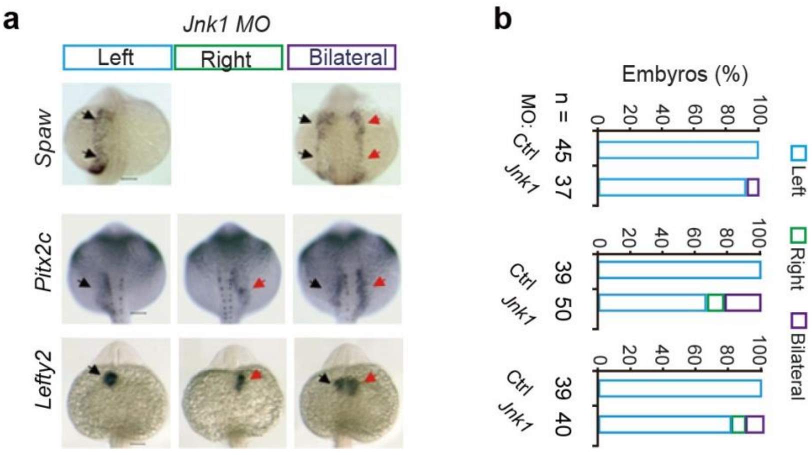

Supplementary Figure 19. *Jnk1* depletion-resulted in randomized expression of left side specific *spaw* and its target genes. Embryos were injected with *jnk1* or control MO at the 1-4-cell stage and harvested at the 10s stage. (**a**) Randomized expression of left side-specific *spaw* and its target genes of *pitx2c, lefty1* and *lefty2* in *jnk1* morphants. Scale bar, 100 μm. (**b**) Quantitative analysis of embryos with normal (left-sided), reversed (right-sided), and bilateral expression.

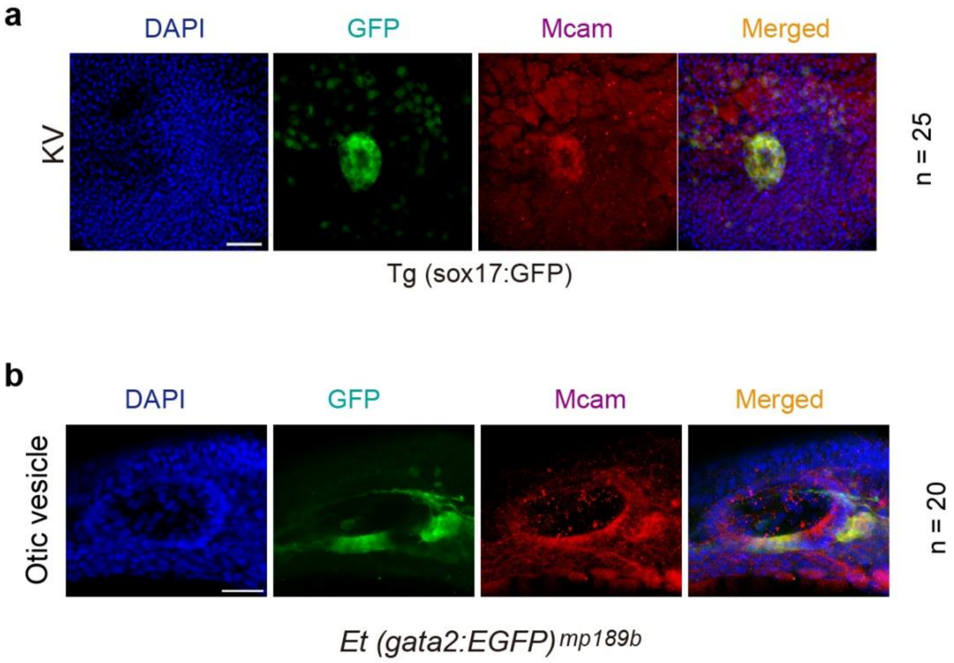

Supplementary Figure 20. Endogenous MCAM is localized at KV and otic vesicle. **(a)** Embryos of Tg (sox17:GFP) zebrafish line was harvested at the 28 hpf stage and the KV was stained with MCAM antibody. **(b)** Embryos of Et (gata2:EGFP) ^{mp189b} zebrafish line was harvested at the 28 hpf stage and the otic vesicle was stained with MCAM antibody. The nucleus was stained with DAPI. Scale bars: 30 μm in **a**; 50 μm in **b**.

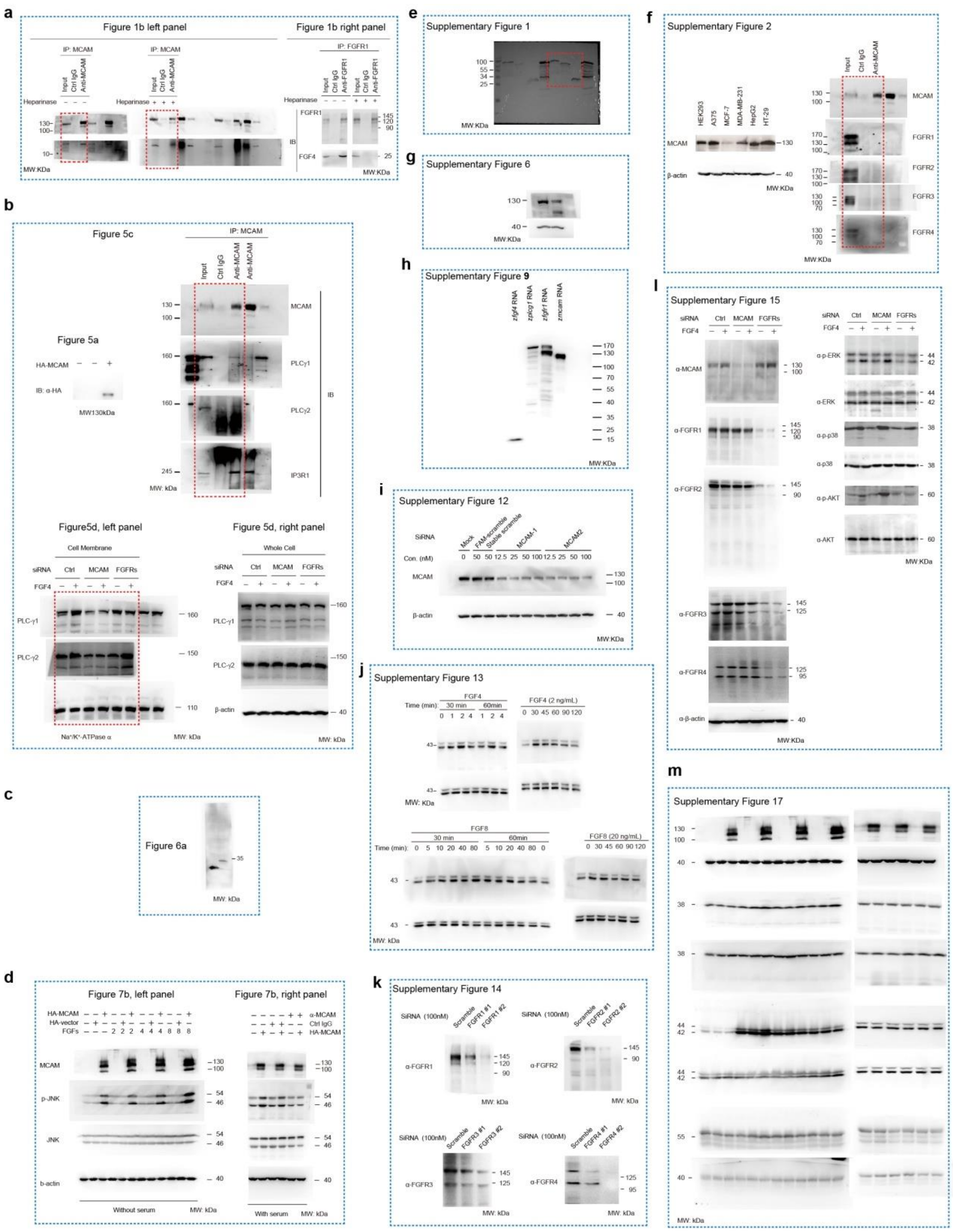

Supplementary Figure 21. All uncropped versions of the Western Blots are presented in the same order as in the corresponding figures.
